# One-pot four-component synthesis of novel isothiourea-ethylene-tethered-piperazine derivatives†

**DOI:** 10.1039/d3ra06678a

**Published:** 2023-11-07

**Authors:** Fatima Hajizadeh, Mohammad M. Mojtahedi, M. Saeed Abaee

**Affiliations:** a Organic Chemistry Department, Chemistry and Chemical Engineering Research Center of Iran P.O. Box 14335-186 Tehran Iran mojtahedi@ccerci.ac.ir

## Abstract

An efficient metal-free four-component approach for the synthesis of piperazine derivatives tethered to an isothiourea group through an ethylene link was developed. 1,4-Diazabicyclo[2.2.2]octane (DABCO) salts, generated *in situ* through the reactions of DABCO with various alkyl bromides, reacted with phenylisothiocyanate (PITC) and amines in a one-pot manner to give the target products. Initially, through two parallel nucleophilic paths, DABCO and the secondary amine adds to the alkyl bromide and PITC, respectively. The process is followed by the combination of the two respective intermediates to produce the final products by forming a new C–S bond with the expense of a C–N bond cleavage. Consequently, various DABCO salts and secondary amines were tolerated well in this protocol to afford the isothiourea-ethylene-tethered-piperazine compounds in good to high yields.

## Abstract 2

A multicomponent synthesis of piperazines tethered to isothiourea group *via* an ethylene link was developed. Several derivatives of the target products are formed by combination of DABCO, alkyl bromides, secondary amines, and phenylisothiocyanate.

## Graphical abstract

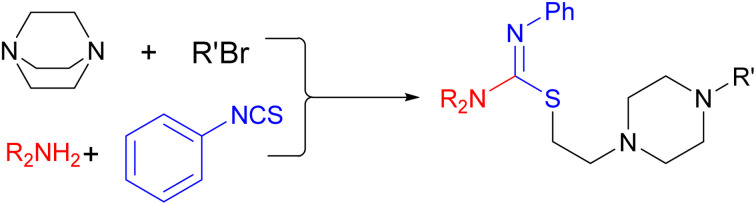

## Introduction

Multicomponent reactions (MCRs) have emerged as a promising synthetic strategy in organic chemistry in recent decades, since they furnish one-pot routes to convert commercially available reactants to complex structures.^1,2^ In addition, MCRs are attractive from economical, operational simplicity, and green chemistry points of view.^3,4^ Consequently, MCRs are widely employed in the synthesis of various natural products,^5^ heterocyclic systems,^6^ and other molecules of interest.^7–9^ However, earlier MCRs often involved the use of limited starting materials, higher reaction temperatures, and toxic reagents. Thus, there is an ongoing demand for further improvement of MCRs by designing new processes with lower overall costs, better selectivity, higher efficiency, enhanced environmental aspects, and improved atom-economy.

**Fig. 1:**
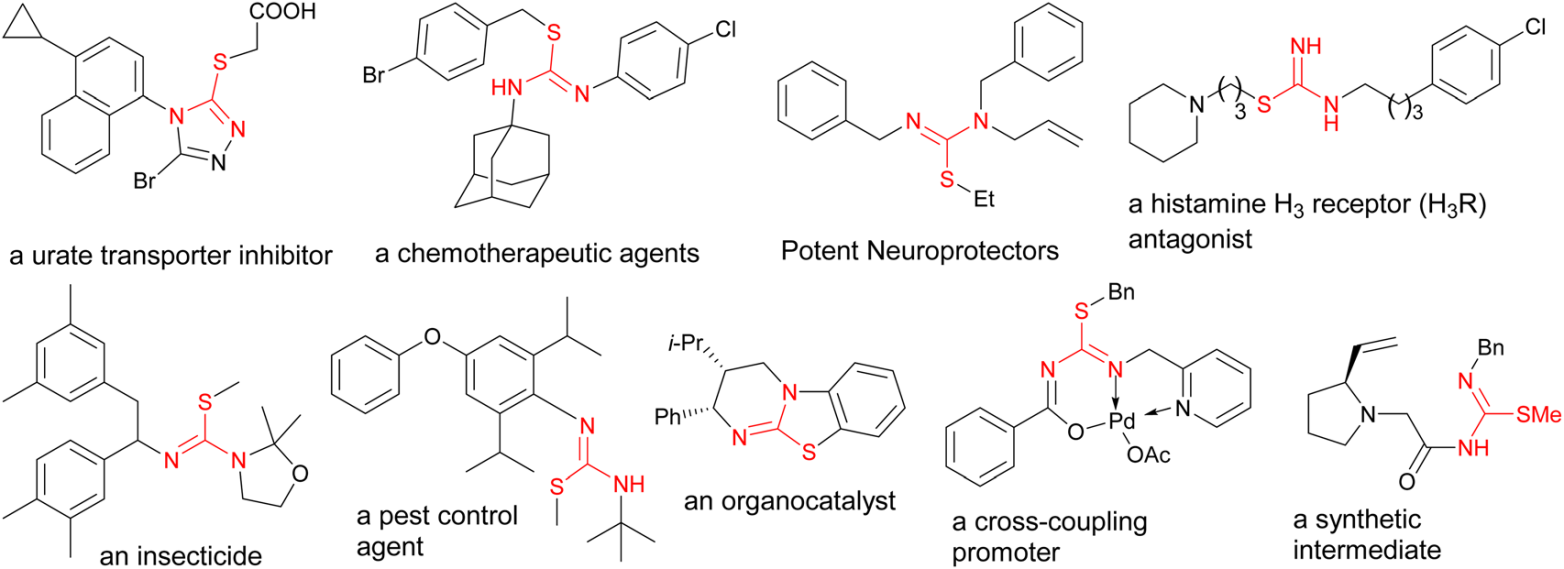
Important molecules containing the isothiourea functional group.

Molecules containing isothiourea moieties constitute important structures in medicinal,^10,11^ biological,^12,13^ and agricultural chemistry.^14,15^ In addition, they are also employed as catalysts^16,17^ or reactive intermediates^18^ in other synthetic procedures. Illustrative related structures are highlighted in Fig. 1. Various methods are reported so far for the synthesis of these molecules. The majority of these reports are carried out using conventional stepwise approaches,^19,20^ while a few recent studies deal with three-component procedures. For instance, Sun *et al.* developed a three-component synthesis of isothioureas *via* the combination of isocyanides and amines with disulfides, where the latter component was initially activated by *N*-halogen succinimides using (2,2,6,6-tetramethylpiperidin-1-yl)oxyl (TEMPO).^21^ Alternatively, Maes devised a copper(i) catalyzed three-component reaction between thiosulfonates, amines, and isocyanides, resulting in the synthesis of isothiourea derivatives.^22^ Other important related reports include a tandem process by Wu,^23^ a three-component reaction by Mishra,^24^ and a binuclear aluminium complex mediated synthesis of carbodiimides by Panda.^25^

**Scheme 1:**
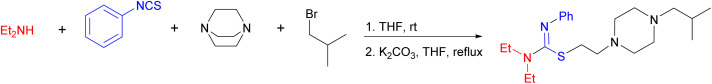
One-pot four-component approach for the synthesis of isothiourea-ethylene-tethered piperazine derivatives.

Despite all the studies carried out on isothioureas, many traditional methods suffer from the use of complicated steps, toxic reagents or additives, and poor reactivity of the starting materials. Therefore, there is a need for further development of efficient and sustainable synthetic methods involving isothiourea chemistry. In the framework of our program on MCRs^26,27^ and heterocyclic chemistry,^28,29^ we would like to report a novel four-component procedure for the synthesis of a new series of isothiourea containing piperazines, as exemplified in Scheme 1 for the reaction of diethylamine (Et_2_NH), phenyl isothiocyanate (PITC), 1,4-diazabicyclo[2.2.2]octane (DABCO), and 1-bromo-2-methylpropane.

Although the use of DABCO bond cleavage for the synthesis of various piperazine derivatives has precedence,^30–32^ the current work is the first application of DABCO salts in the synthesis of the isothiourea functional group. For this purpose, we planned to use two very reactive species, the electrophilic isothiocyanate (PITC) moiety and the sterically hindered tertiary amine (DABCO), whose reactive natures can trigger parallel nucleophilic combinations of the reactants, which is a useful tool for launching multicomponent reactions. Consequently, this leads to a concurrent C–N bond cleavage and C–S bond formation reactions to produce the target products, in which the isothiourea and piperazine functional groups are placed in vicinity and would be interesting candidates for further biological studies.

## Results and discussion

For simplicity, we first synthesized the DABCO salts 2 separately to start the study with a three-component process. Thus, to optimize the reaction, we subjected Et_2_NH 1a and PITC to combine with 2a under various conditions (Table 1). Treatment of a 1.0 : 1.0 : 1.0 mixture of the three reactants and K_2_CO_3_ at refluxing temperature in THF after 5 h led to the formation of 3a in 93% yield (entry 1). In the absence of the base (entry 2) or at lower temperatures (entries 3–4), the yield was diminished even at a longer reaction time. Similarly, use of other inorganic bases (entries 5–8) did not led to higher conversion of the reactants to 3a. Alternatively, no better results were achieved for conducting the reaction in other protic (entries 9–11) or aprotic (entries 12–16) solvents, conveying that K_2_CO_3_/THF/reflux conditions would provide the highest conversion of the reactants to the desired product.

**Table 1:** Three-component optimization of the synthesis of 3a

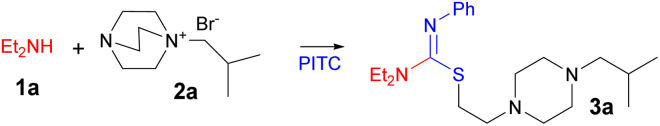					
Entry	Base	Solvent	*T* (°C)	Time (h)	Yield^a^ (%)
1	K_2_CO_3_	THF	Reflux	5	93
2	—	THF	Reflux	24	28
3	K_2_CO_3_	THF	50	24	49
4	K_2_CO_3_	THF	25	24	20
5	Na_2_CO_3_	THF	Reflux	24	59
6	K_3_PO_4_	THF	Reflux	24	54
7	KOH	THF	Reflux	24	39
8	NaOH	THF	Reflux	24	37
9	K_2_CO_3_	EtOH	Reflux	24	64
10	K_2_CO_3_	MeOH	Reflux	24	70
11	K_2_CO_3_	H_2_O	Reflux	24	16
12	K_2_CO_3_	DMF	Reflux	24	37
13	K_2_CO_3_	DMSO	Reflux	24	<5
14	K_2_CO_3_	CHCl_3_	Reflux	24	26
15	K_2_CO_3_	CH_2_Cl_2_	Reflux	24	<5
16	K_2_CO_3_	*n*-Hexane	Reflux	24	18

a Isolated yields.

With these results in hand, next we extended the process into a four-component combination by primarily subjecting DABCO to react with various alkyl bromides to provide the required salts 2 for the following steps (Table 2). Consequently, when DABCO, Me_2_CHCH_2_Br, Et_2_NH, and PITC were reacted in this manner, 3a was produced after 12 h in 91% yield (entry 1). By using this approach, Et_2_NH and PITC reacted with other *in situ* generated derivatives of 2 to give 3b–e efficiently (entries 2–5). Similarly, products 3f–j were obtained in the same manner to emphasize the generality of the process (entries 6–10).

**Table 2:** Diversity scope of the process by 4-component synthesis of derivatives of 3^a^^,^^b^

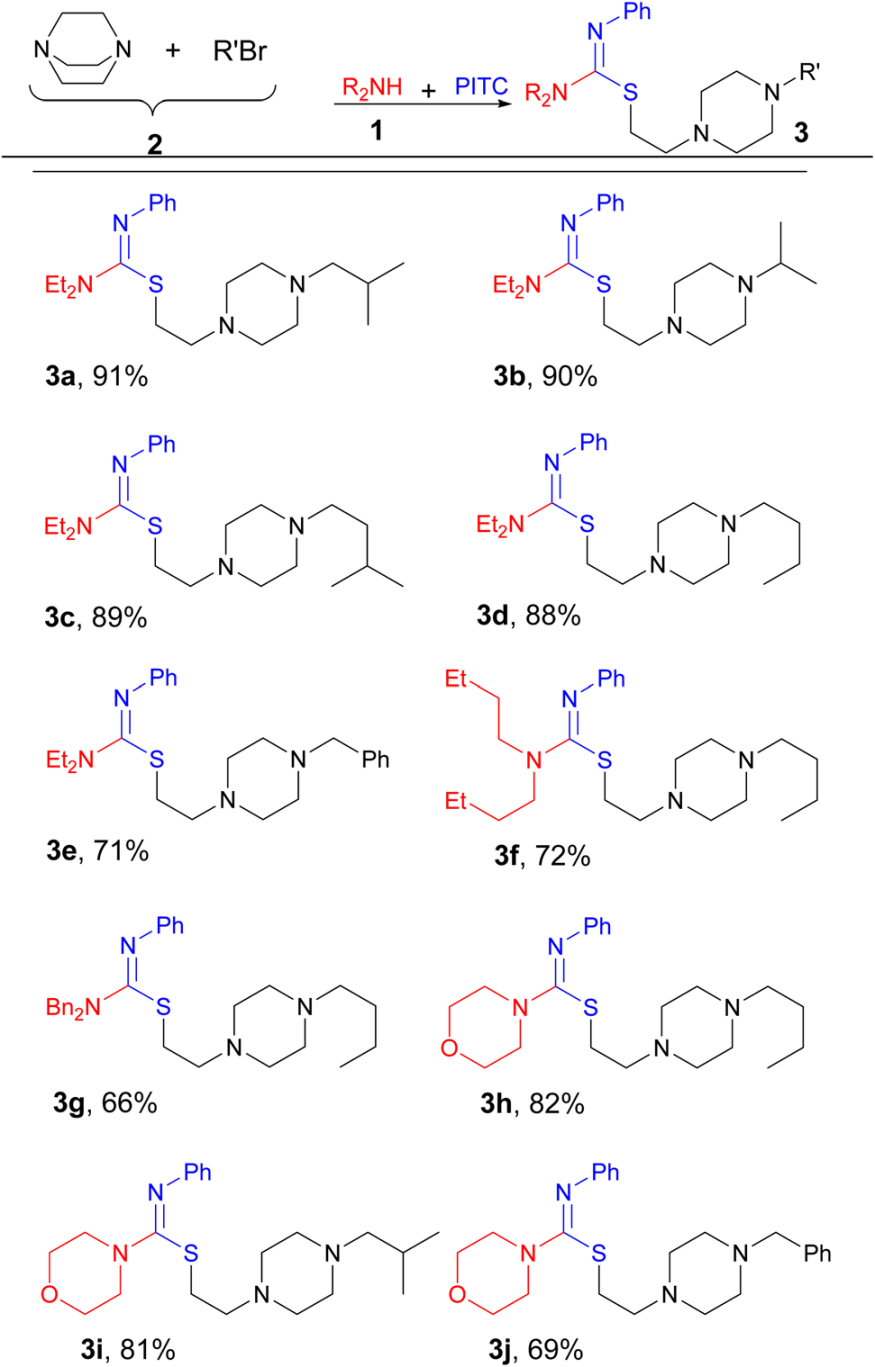

a Reaction conditions: DABCO (10.0 mmol), THF (30 mL) R′Br (10.0 mmol), rt, 10–12 h. Then added to this mixture R_2_NH (10.0 mmol), PITC (10.0 mmol), K_2_CO_3_ (10.0 mmol), THF (10 mL) reflux 4–6 h.

b Isolated yields.

**Fig. 2:**
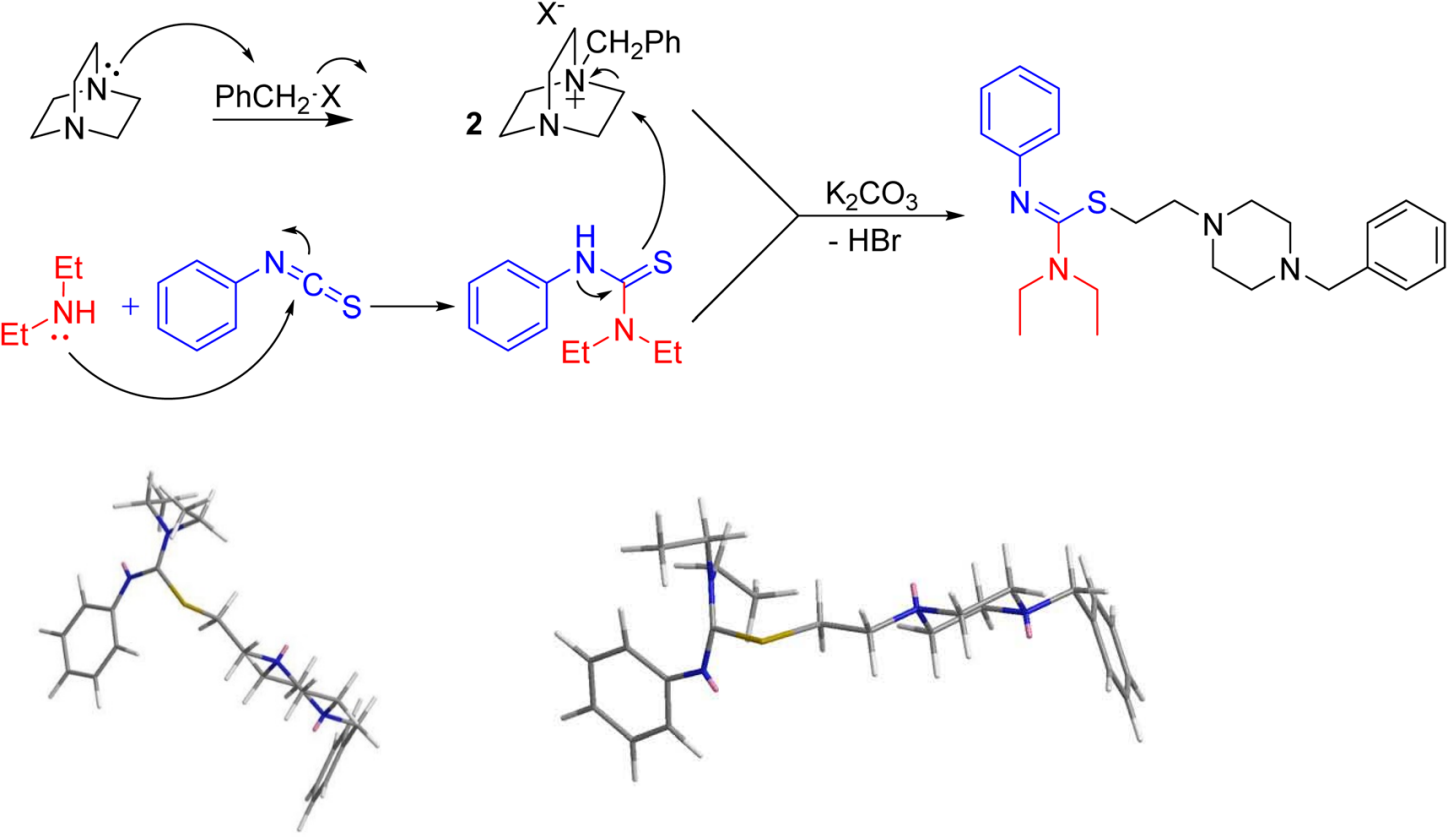
Proposed mechanism for the synthesis of the products (top) and configurational projection of MM2-energy-minimised 3e (bottom) obtained from ChemDraw Pro.

Based on these results, a mechanism would be proposed for this process. Initially, DABCO is alkylated *via* a nucleophilic attack on the alkyl bromide moiety to produce 2, while in a parallel reaction, Et_2_NH adds to PITC. The two resulting intermediates of the initial steps then would combine through K_2_CO_3_-activated attack of the diethyl-phenylthiourea species to the DABCO salt to access to the final products. The stereochemistry of the isothiourea functional group was assigned as *Z* (as seen in Fig. 2 (top) for the product with R = Et and R′ = CH_2_Ph). Such assignment for similar molecules resulting from the same chemistry is reported before in the literature.^15,33^ To further support the assignment, we engaged molecular mechanics calculations to verify the proposed stereochemistry. The results arising from molecular mechanics (MM2) calculations using ChemSoft's ChemOffice Pro (version 20) clearly show that the *Z* configuration (Fig. 2, bottom-left) is more stable that the *E* counterpart (Fig. 2, bottom-right).

## Experimental

### General

FT-IR spectra were recorded using KBr disks on a Bruker Vector-22 spectrometer. NMR spectra were obtained on a Bruker AMX (300 MHz for ^1^H and 75 MHz for ^13^C) as CDCl_3_ solutions using TMS as internal standard reference. Elemental analyses were performed using a Thermo Finnigan Flash EA 1112 instrument. MS spectra were obtained on a Fisons 8000 Trio instrument at ionization potential of 70 eV. TLC experiments were carried out on pre-coated silica gel plates using petroleum ether/EtOAc as the eluent. Starting materials and reagents were purchased from commercial sources.

### Synthesis of ammonium salts 2a from DABCO

To a solution of DABCO (1.12 g, 10.0 mmol) in THF (20 mL) was added Me_2_CHCH_2_Br (10.0 mmol), and the solution was stirred at room temperature for 12 h. A precipitate was formed, which was filtered, washed with ethyl acetate (10 mL) and dried under vacuum to get 2a.

### Typical procedure for three-component synthesis of functionalized piperazines 3a

A mixture of 2a (0.11 g, 0.5 mmol), Et_2_NH (57.0 μL, 0.55 mmol), PITC (0.07 g, 0.5 mmol), and K_2_CO_3_ (0.069 g, 0.5 mmol) in THF (5 mL) was stirred at refluxing temperature for 5 h. After completion of the reaction, the solvent was evaporated under vacuum and the concentrated crude mixture was fractionated by column chromatography on silica gel (EtOAc/petroleum ether; 60/40) to afford the pure product. The isolated product was fully characterized by various spectroscopic methods.

### Typical procedure for four-component synthesis of functionalized piperazines 3a

To a solution of DABCO (1.12 g, 10.0 mmol) in THF (30 mL) was added Me_2_CHCH_2_Br (10.0 mmol), and the solution was stirred at room temperature for 12 h to get 2a. To this was added Et_2_NH (1140 μL, 10.0 mmol), PITC (1.4 g, 10.0 mmol), and K_2_CO_3_ (1.4 g, 10.0 mmol) in THF (10 mL) and the mixture was stirred at refluxing temperature for 5 h. After completion of the reaction, the solvent was evaporated under vacuum and the concentrated crude mixture was fractionated by column chromatography on silica gel (EtOAc/petroleum ether; 60/40) to afford the pure product. The isolated product was fully characterized by various spectroscopic methods.

### 2-(4-Isobutylpiperazin-1-yl)ethyl (*Z*)-*N*,*N*-diethyl-*N*′-phenylcarbamimidothioate 3a

^1^H NMR (300 MHz, CDCl_3_) *δ* 7.21–7.16 (m, 2H), 6.92–6.86 (m, 3H), 3.54 (q, *J* = 7.0 Hz, 4H), 2.47–2.41 (m, 2H), 2.38–2.29 (m, 10H), 2.04 (d, *J* = 7.0 Hz, 2H), 1.70 (t, septet, *J* = 7.0, 6.5 Hz, 1H), 1.17 (t, *J* = 7.0 Hz, 6H), 0.85 (d, *J* = 6.5 Hz, 6H) ppm; ^13^C NMR (75 MHz, CDCl_3_) *δ* 152.3, 149.8, 128.3, 121.4, 121.2, 66.5, 57.4, 53.1, 52.4, 44.0, 28.6, 25.0, 20.7, 20.6, 13.5 ppm; IR (KBr) *ν* = 2953, 2807, 1568, 1227, 1112 cm^−1^; MS (70 eV) *m*/*z* 376 [M^+^], 235, 168, 125; anal. calcd for C_21_H_36_N_4_S: C, 66.97; H, 9.64; N, 14.88; S, 8.51. Found: C, 66.76; H, 9.80; N, 14.97; S, 8.67.

### 2-(4-Isopropylpiperazin-1-yl)ethyl (*Z*)-*N*,*N*-diethyl-*N*′-phenylcarbamimidothioate 3b

^1^H NMR (300 MHz, CDCl_3_) *δ* 7.29–7.23 (m, 2H), 6.97–6.91 (m, 3H), 3.53 (q, *J* = 7.0 Hz, 4H), 2.78 (septet, *J* = 6.5 Hz, 1H), 1.69–1.58 (m, 4H), 1.43–1.36 (m, 8H), 1.10 (d, *J* = 6.5 Hz, 6H), 0.99 (t, *J* = 7.0 Hz, 6H) ppm; ^13^C NMR (75 MHz, CDCl_3_) *δ* 153.2, 150.3, 128.6, 121.5, 121.1, 68.1, 49.8, 48.7, 30.7, 29.7, 29.6, 20.1, 14.0 ppm; IR (KBr) *ν* = 2954, 2809, 1577, 1227 cm^−1^; MS (70 eV) *m*/*z* 362 [M^+^], 279, 248, 192; anal. calcd for C_20_H_34_N_4_S: C, 66.25; H, 9.45; N, 15.45; S, 8.84. Found: C, 66.14; H, 9.23; N, 15.60; S, 9.05.

### 2-(4-Isopentylpiperazin-1-yl)ethyl (*Z*)-*N*,*N*-diethyl-*N*′-phenylcarbamimidothioate 3c

^1^H NMR (300 MHz, CDCl_3_) *δ* 7.21–7.15 (m, 2H), 6.94–6.84 (m, 3H), 3.53 (q, *J* = 7.0 Hz, 4H), 2.45–2.40 (m, 4H), 2.38–2.33 (m, 4H), 2.31–2.24 (m, 6H), 1.53 (t, septet, *J* = 7.0, 6.5 Hz, 1H), 1.36–1.29 (m, 2H), 1.16 (t, *J* = 7.0 Hz, 6H), 0.86 (d, *J* = 6.5 Hz, 6H) ppm; ^13^C NMR (75 MHz, CDCl_3_) *δ* 152.5, 149.9, 128.4, 121.4, 121.1, 57.4, 56.7, 53.0, 52.4, 44.1, 35.6, 28.7, 26.5, 22.6, 13.7 ppm; IR (KBr) *ν* = 2953, 2808, 1578, 1227, 1112 cm^−1^; MS (70 eV) *m*/*z* 390 [M^+^], 350, 318, 214, 182; anal. calcd for C_22_H_38_N_4_S: C, 67.64; H, 9.81; N, 14.34; S, 8.21. Found: C, 67.82; H, 10.08; N, 14.58; S, 8.05.

### 2-(4-Butylpiperazin-1-yl)ethyl (*Z*)-*N*,*N*-diethyl-*N*′-phenylcarbamimidothioate 3d

^1^H NMR (300 MHz, CDCl_3_) *δ* 7.21–7.15 (m, 2H), 6.92–6.86 (m, 3H), 3.54 (q, *J* = 7.0 Hz, 4H), 2.48–2.36 (m, 4H), 2.36–2.19 (m, 10H), 1.47–1.37 (m, 2H), 1.33–1.27 (m, 2H), 1.17 (t, *J* = 7.0 Hz, 6H), 0.88 (t, *J* = 7.0 Hz, 3H) ppm; ^13^C NMR (75 MHz, CDCl_3_) *δ* 152.6, 149.9, 128.4, 121.5, 121.3, 58.3, 57.4, 52.9, 52.4, 44.1, 28.8, 28.7, 20.6, 13.8, 13.6 ppm; IR (KBr) *ν* = 2957, 2808, 1577, 1227 cm^−1^; MS (70 eV) *m*/*z* 376 [M^+^], 264, 168, 125; anal. calcd for C_21_H_36_N_4_S: C, 66.97; H, 9.64; N, 14.88; S, 8.51. Found: C, 66.77; H, 9.83; N, 14.99; S, 8.43.

### 2-(4-Benzylpiperazin-1-yl)ethyl (*Z*)-*N*,*N*-diethyl-*N*′-phenylcarbamimidothioate 3e

^1^H NMR (300 MHz, CDCl_3_) *δ* 7.36–7.30 (m, 5H), 7.25–7.20 (m, 2H), 6.95–6.93 (m, 3H), 3.58 (q, *J* = 7.0 Hz, 4H), 3.52 (s, 2H), 2.57–2.37 (m, 12H), 1.21 (t, *J* = 7.0 Hz, 6H) ppm; ^13^C NMR (75 MHz, CDCl_3_) *δ* 152.8, 150.0, 129.3, 128.7, 128.2, 127.1, 121.7, 121.5, 121.3, 62.9, 57.5, 52.7, 52.4, 44.3, 28.8, 13.7 ppm; IR (KBr) *ν* = 2931, 2807, 1578, 1227 cm^−1^; MS (70 eV) *m*/*z* 410 [M^+^], 264, 202, 175, 146; anal. calcd for C_24_H_34_N_4_S: C, 70.20; H, 8.35; N, 13.64; S, 7.81. Found: C, 70.41; H, 8.57; N, 13.49; S, 7.95.

### 2-(4-Butylpiperazin-1-yl)ethyl (*Z*)-*N*,*N*-dibutyl-*N*′-phenylcarbamimidothioate 3f

^1^H NMR (300 MHz, CDCl_3_) *δ* 7.22–7.17 (m, 2H), 6.91–6.87 (m, 3H), 3.48 (t, *J* = 7.5 Hz, 4H), 2.46–2.41 (m, 4H), 2.36–2.35 (m, 10H), 1.62–1.52 (m, 4H), 1.48–1.36 (m, 2H), 1.34–1.22 (m, 6H), 0.92 (t, *J* = 7.5 Hz, 3H), 0.89 (t, *J* = 7.0 Hz, 6H) ppm; ^13^C NMR (75 MHz, CDCl_3_) *δ* 153.1, 150.0, 128.5, 121.5, 121.2, 58.3, 57.4, 53.0, 52.4, 49.7, 30.5, 28.8, 28.7, 20.6, 19.9, 13.9, 13.8 ppm; IR (KBr) *ν* = 2930, 1578, 1376, 1161 cm^−1^; MS (70 eV) *m*/*z* 432 [M^+^], 261, 207, 168, 125; anal. calcd for C_25_H_44_N_4_S: C, 69.39; H, 10.25; N, 12.95; S, 7.41. Found: C, 69.19; H, 10.41; N, 13.08; S, 7.57.

### 2-(4-Benzylpiperazin-1-yl)ethyl (*Z*)-*N*,*N*-dibutyl-*N*′-phenylcarbamimidothioate 3g

^1^H NMR (300 MHz, CDCl_3_) *δ* 7.34–7.30 (m, 5H), 7.25–7.19 (m, 3H), 6.95–6.89 (m, 3H), 3.52 (s, 2H), 3.51 (t, *J* = 7.5 Hz, 3H), 2.53–2.45 (m, 2H), 2.45–2.35 (m, 10H), 1.60 (t, t, *J* = 7.5, 7.5 Hz, 4H), 1.32 (t, q, *J* = 7.0, 7.5 Hz, 4H), 0.95 (t, *J* = 7.0 Hz, 6H) ppm; ^13^C NMR (75 MHz, CDCl_3_) *δ* 153.3, 150.1, 134.0, 129.3, 128.6, 128.2, 127.1, 121.6, 121.4, 62.9, 57.5, 52.7, 52.4, 49.9, 30.7, 20.1, 14.0 ppm; IR (KBr) *ν* = 2941, 2808, 1578, 1159 cm^−1^; MS (70 eV) *m*/*z* 466 [M^+^], 320, 265, 202, 146; anal. calcd for C_28_H_42_N_4_S: C, 72.06; H, 9.07; N, 12.00; S, 6.87. Found: C, 71.90; H, 9.15; N, 12.11; S, 6.97.

### 2-(4-Butylpiperazin-1-yl)ethyl (*Z*)-*N*-phenylmorpholine-4-carbimidothioate 3h

^1^H NMR (300 MHz, CDCl_3_) *δ* 7.21 (d, t, *J* = 7.5, 2.0 Hz, 2H), 6.94 (t, t, *J* = 7.5, 2.0 Hz, 1H), 6.87–6.84 (m, 2H), 3.69 (t, *J* = 4.5 Hz, 4H), 3.59 (t, *J* = 4.5 Hz, 4H), 2.53–2.47 (m, 4H), 2.38–2.34 (m, 6H), 2.28–2.24 (m, 4H), 1.46–136 (m, 2H), 1.27 (t, q, *J* = 7.0, 7.5 Hz, 2H), 0.87 (t, *J* = 7.0 Hz, 3H) ppm; ^13^C NMR (75 MHz, CDCl_3_) *δ* 154.9, 149.2, 128.4, 121.3, 121.0, 66.4, 58.2, 57.6, 52.8, 52.4, 48.5, 28.9, 28.7, 20.5, 13.9 ppm; IR (KBr) *ν* = 2955, 2807, 1582, 1158 cm^−1^; MS (70 eV) *m*/*z* 390 [M^+^], 336, 281, 168, 125; anal. calcd for C_21_H_34_N_4_OS: C, 64.58; H, 8.77; N, 14.34; S, 8.21. Found: C, 64.62; H, 8.91; N, 14.27; S, 8.40.

### 2-(4-Isobutylpiperazin-1-yl)ethyl (*Z*)-*N*-phenylmorpholine-4-carbimidothioate 3i

^1^H NMR (300 MHz, CDCl_3_) *δ* 7.24 (d, t, 7.5, 1.5 Hz, 2H), 7.25 (d, t, 7.5, 1.5 Hz, 1H), 6.81–6.88 (m, 2H), 3.71 (t, 4.5Hz, 4H), 3.63 (t, 4.5Hz, 4H), 2.56–2.51 (m, 2H), 2.52–2.34 (m, 10H), 2.05 (d, *J* = 7.0 Hz, 2H), 1.77 (t, septet, *J* = 7.0, 6.5 Hz, 1H), 0.88 (d, *J* = 6.5 Hz, 6H) ppm; ^13^C NMR (75 MHz, CDCl_3_) *δ* 154.8, 149.2, 128.3, 121.9, 121.0, 66.5, 66.3, 57.6, 53.0, 52.5, 48.4, 28.9, 25.0, 20.6 ppm; IR (KBr) *ν* = 2953, 2807, 1583, 1159 cm^−1^; MS (70 eV) *m*/*z* 390 [M^+^], 249, 189, 168, 125; anal. calcd for C_21_H_34_N_4_OS: C, 67.58; H, 8.77; N, 14.34; S, 8.21. Found: C, 67.65; H, 8.89; N, 14.47; S, 8.39.

### 2-(4-Benzylpiperazin-1-yl)ethyl (*Z*)-*N*-phenylmorpholine-4-carbimidothioate 3j

^1^H NMR (300 MHz, CDCl_3_) *δ* 7.35–7.31 (m, 5H), 7.29–7.24 (m, 2H), 7.02 (dd, *J* = 7.0, 1.5 Hz, 1H), 6.92–6.90 (m, 2H), 3.76 (t, *J* = 4.5 Hz, 4H), 3.65 (t, *J* = 4.5 Hz, 4H), 3.53 (s, 2H), 2.59–2.54 (m, 2H), 2.49–2.40 (m, 10H) ppm; ^13^C NMR (75 MHz, CDCl_3_) *δ* 155.2, 149.5, 129.3, 128.7, 128.2, 127.1, 122.3, 121.5, 121.3, 66.7, 62.9, 57.8, 52.7, 52.5, 48.7, 29.1 ppm; IR (KBr) *ν* = 2934, 2807, 1581, 1156 cm^−1^; MS (70 eV) *m*/*z* 424 [M^+^], 278, 202, 146; anal. calcd for C_24_H_32_N_4_OS: C, 67.89; H, 7.60; N, 13.20; S, 7.55. Found: C, 67.70; H, 7.81; N, 13.32; S, 7.74.

## Conclusions

In summary, we developed a method for the synthesis of a novel series of isothiourea-ethylene-tethered piperazine derivatives. The process can be performed in the same vessel using all the four required reactants in a one-pot manner. The operation is convenient, the process is multicomponent and atom economy, no expensive reagent is required, and each reaction gives a sole product in good yields. The products are expected to exhibit biological properties and would be assessed accordingly in due course.

## Author contributions

Conceptualization, study design, data validation, supervision, project administration, and funding acquisition: Mohammed M. Mojtahedi; methodology; investigation, resources, software, formal analysis, and data curation: Fatima Hajizadeh; writing, original draft preparation, review and editing: M. Saeed Abaee. All authors have read and agreed to the published version of the manuscript.

## Conflicts of interest

There are no conflicts to declare.

## Supplementary Material

RA-013-D3RA06678A-s001
