# Supplementary material for: One-pot four-component synthesis of novel isothiourea-ethylene-tethered-piperazine derivatives
Source: RSC Adv. 2023 Nov 7;13(46):32772–7. doi: 10.1039/d3ra06678a (PMC10629393; doi:10.1039/d3ra06678a)

Electronic Supplementary Material (ESI) for RSC Advances.
This journal is © The Royal Society of Chemistry 2023

One-pot four-component synthesis of novel isothiourea ethylene-tethered piperazine derivatives

Fatima Hajizadeh, Mohammad M. Mojtahedi,* M. Saeed Abaee

Organic Chemistry Department, Chemistry and Chemical Engineering Research Center of Iran,

P.O. Box 14335-186, Tehran, Iran, e-mail: mojtahedi@ccerci.ac.ir

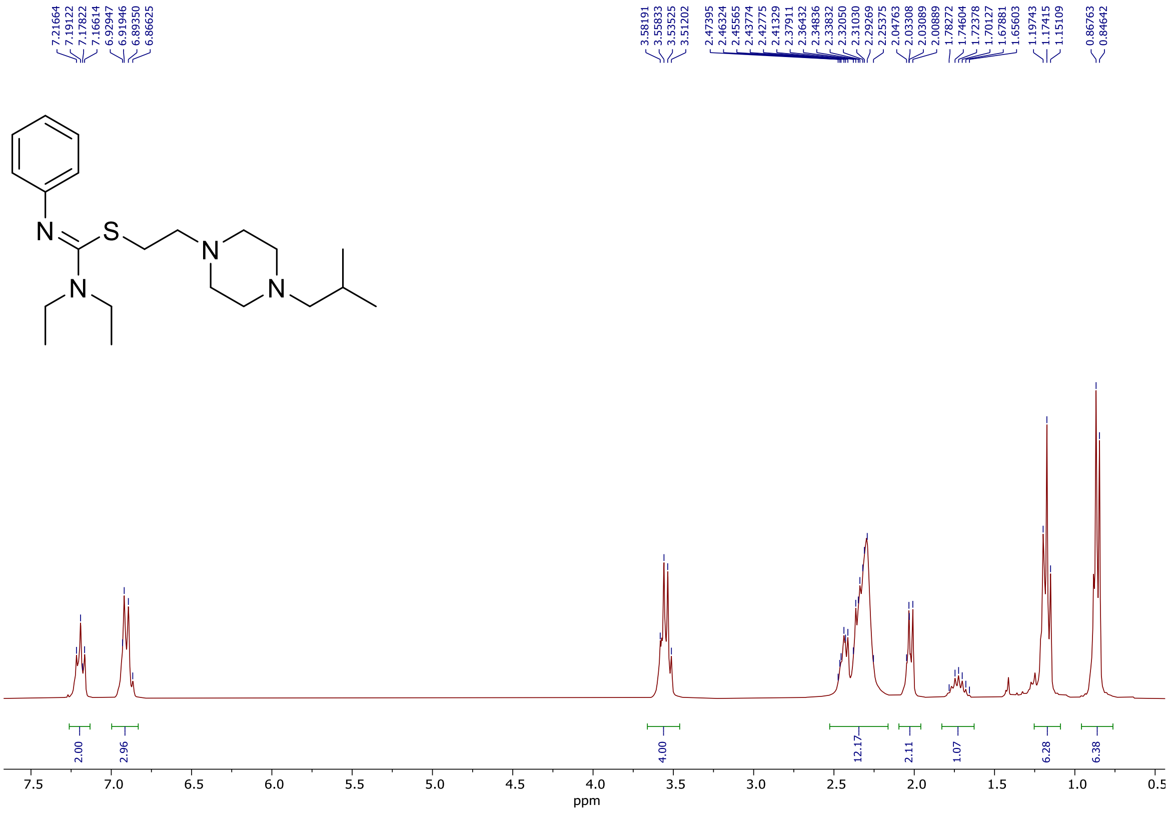

^{1}H NMR (300 MHz, $CDCl_3$); *2-(4-Isobutylpiperazin-1-yl)ethyl (Z)-N,N-diethyl-N'-phenylcarbamimidothioate* **3a**

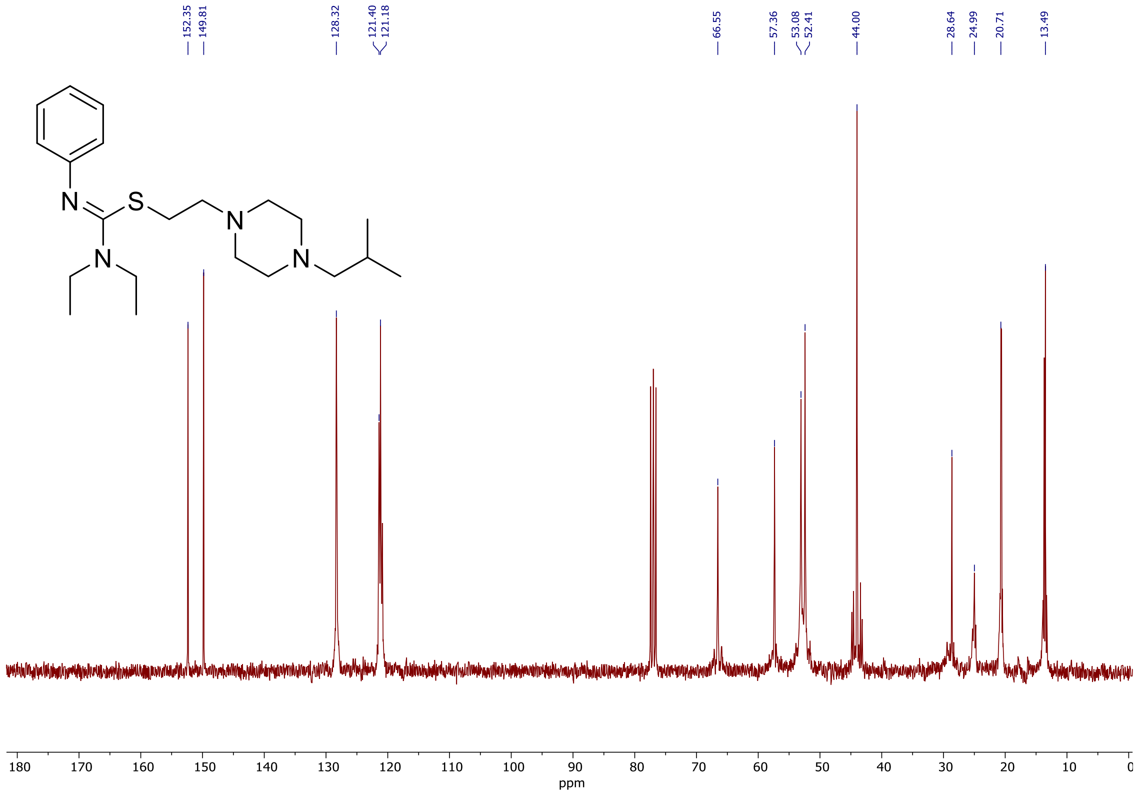

^{13}C NMR (75 MHz, $CDCl_3$); *2-(4-Isobutylpiperazin-1-yl)ethyl (Z)-N,N-diethyl-N'-phenylcarbamimidothioate* **3a**

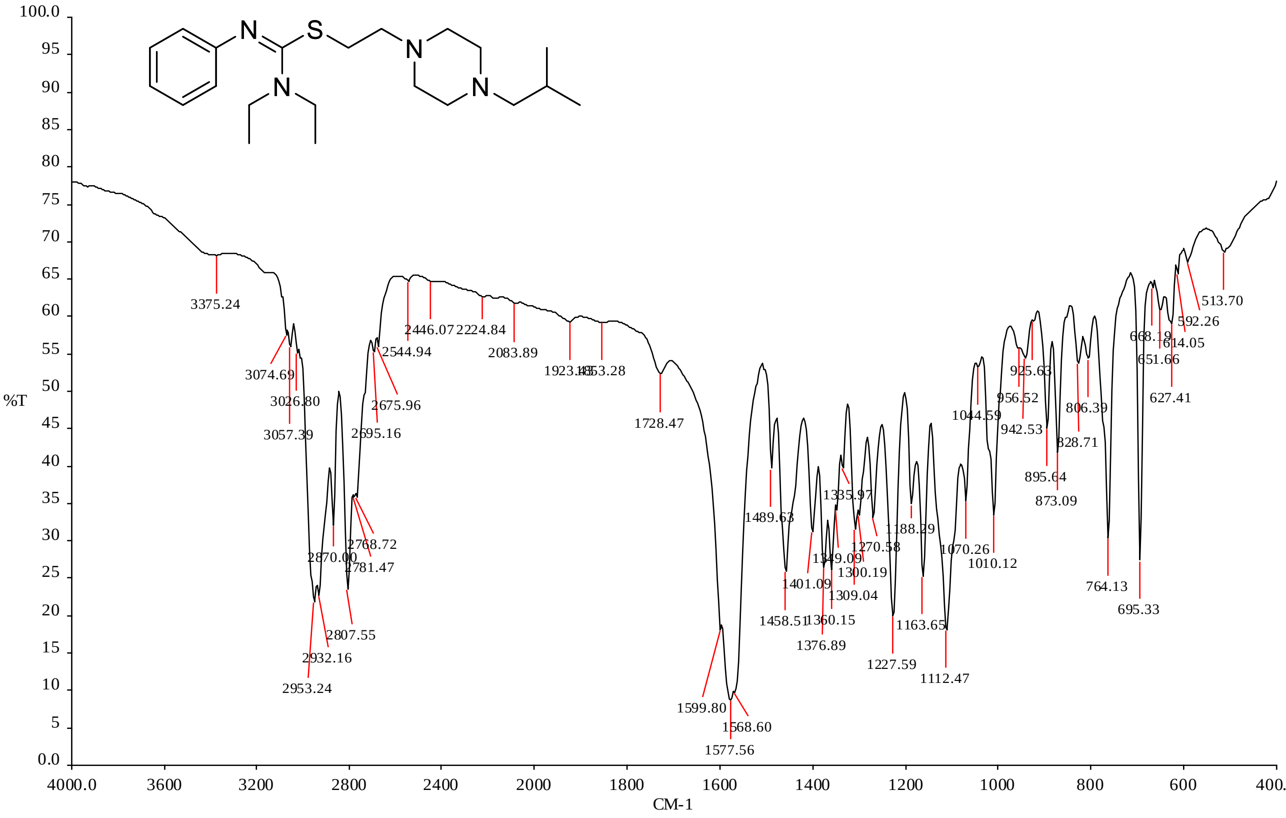

Mr. Moradi **Sample: D 3**

[illegible]
Acquired : 3 Jan 2007 7:06 using AcqMethod f1.M
Instrument : MSD
Sample Name: D3
Misc Info :
Vial Number: 1

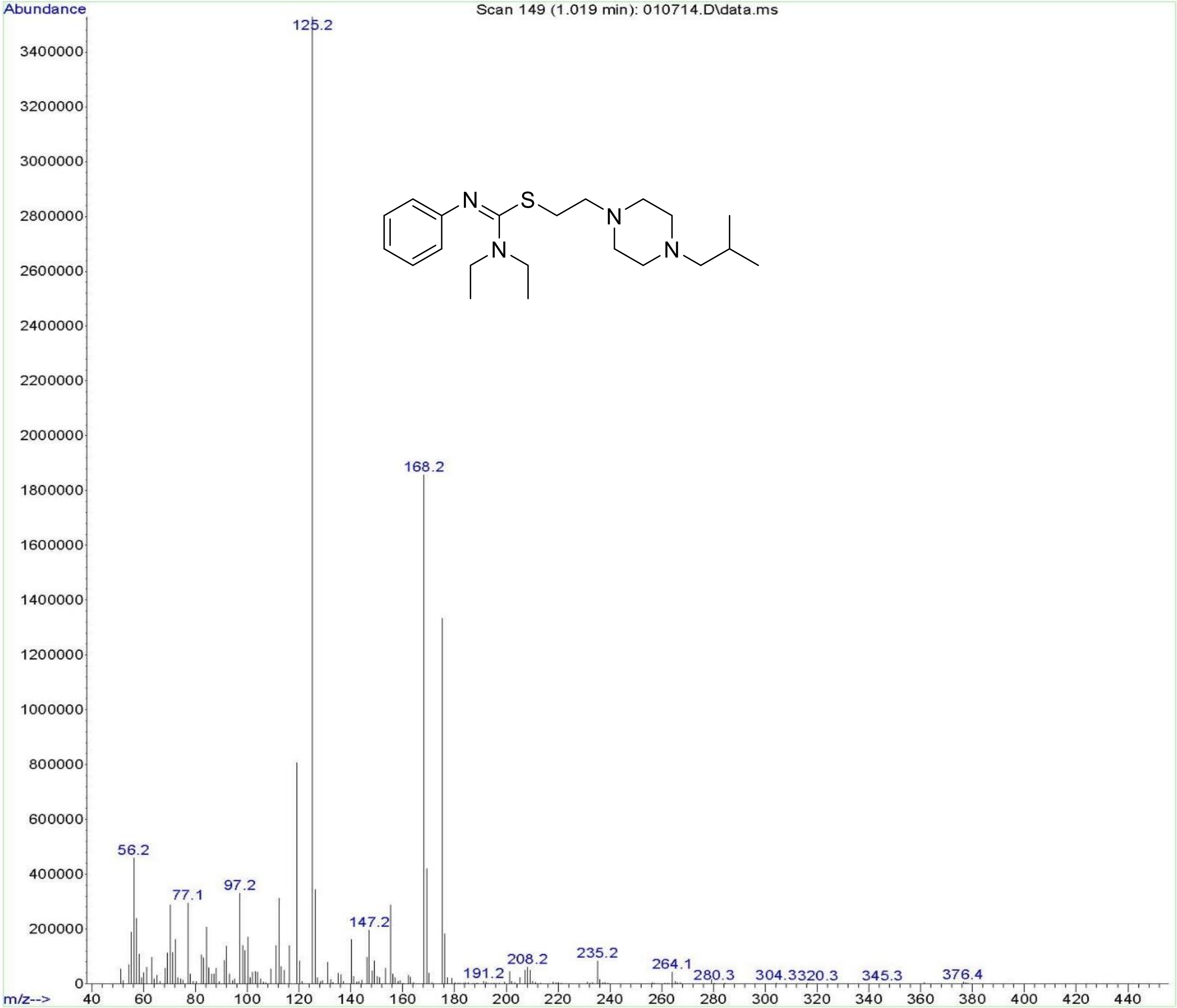

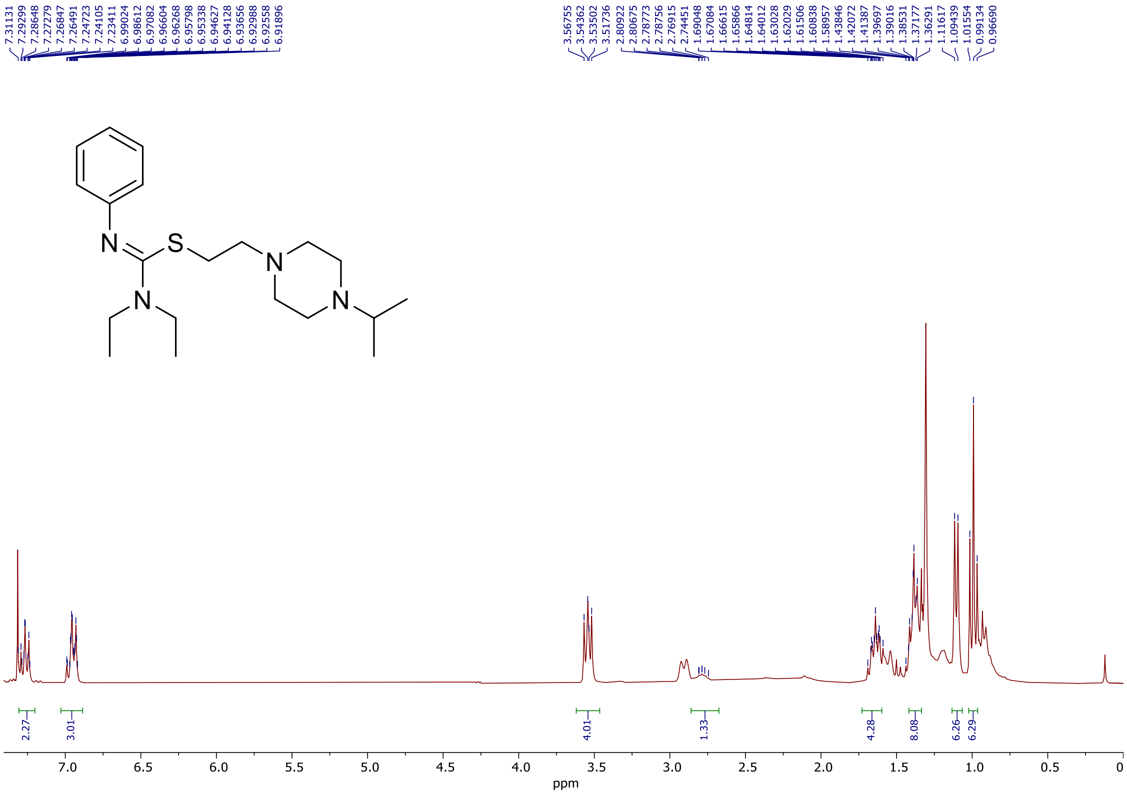

^{1}H NMR (300 MHz, $CDCl_3$); *2-(4-Isopropylpiperazin-1-yl)ethyl (Z)-N,N-diethyl-N'-phenylcarbamimidothioate* **3b**

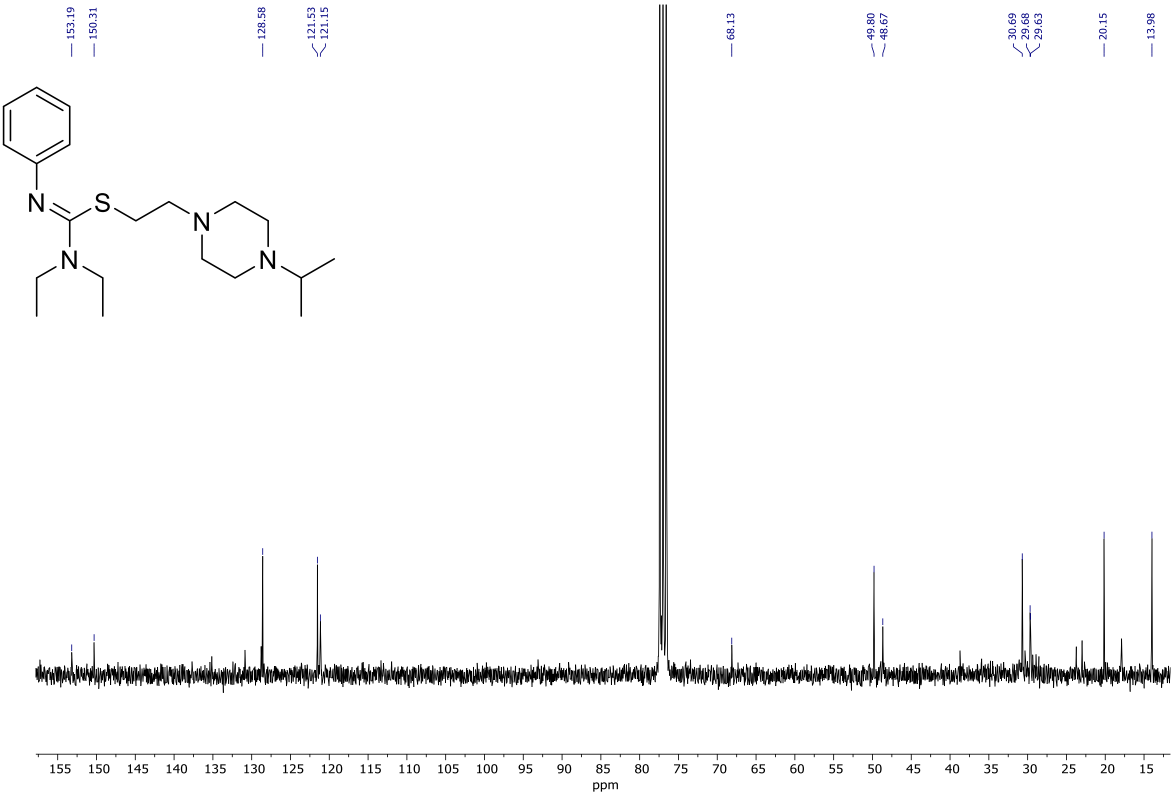

^{13}C NMR (75 MHz, $CDCl_3$); *2-(4-Isopropylpiperazin-1-yl))ethyl (Z)-N,N-diethyl-N'-phenylcarbamimidothioate* **3b**

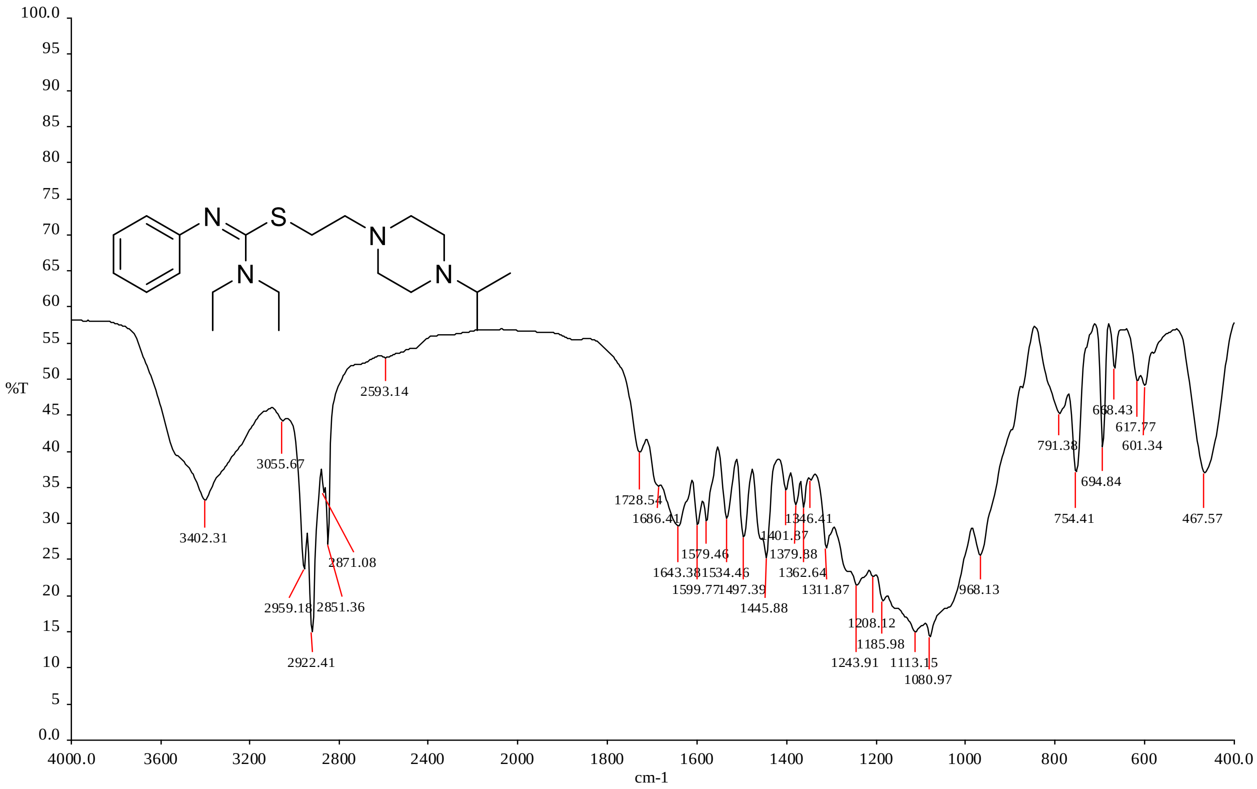

Mr. Moradi **Sample: D5**

[illegible]
Acquired : 3 Jan 2007 6:19 using AcqMethod f1.M
Instrument : MSD
Sample Name: D5
Misc Info :
Vial Number: 1

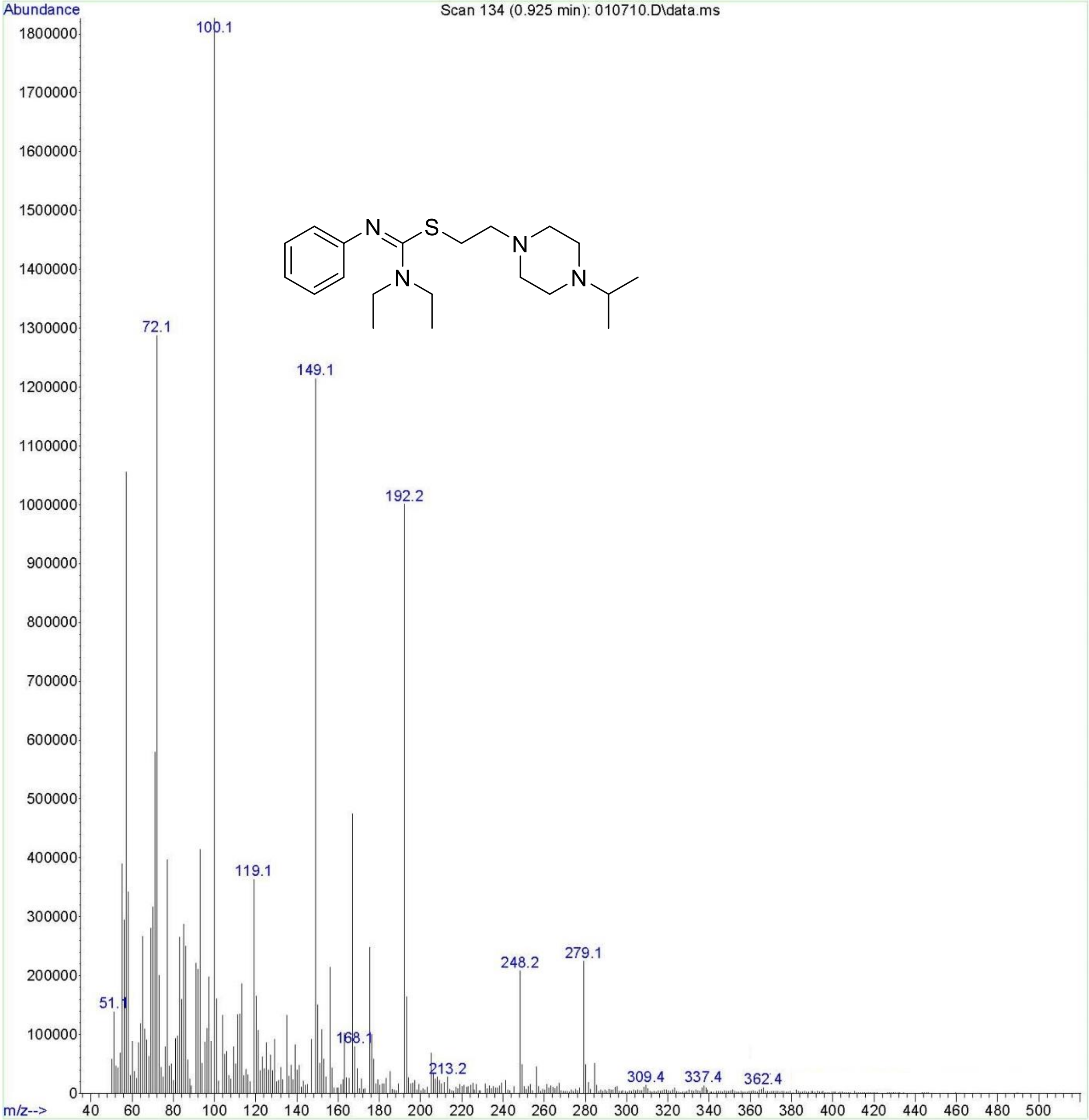

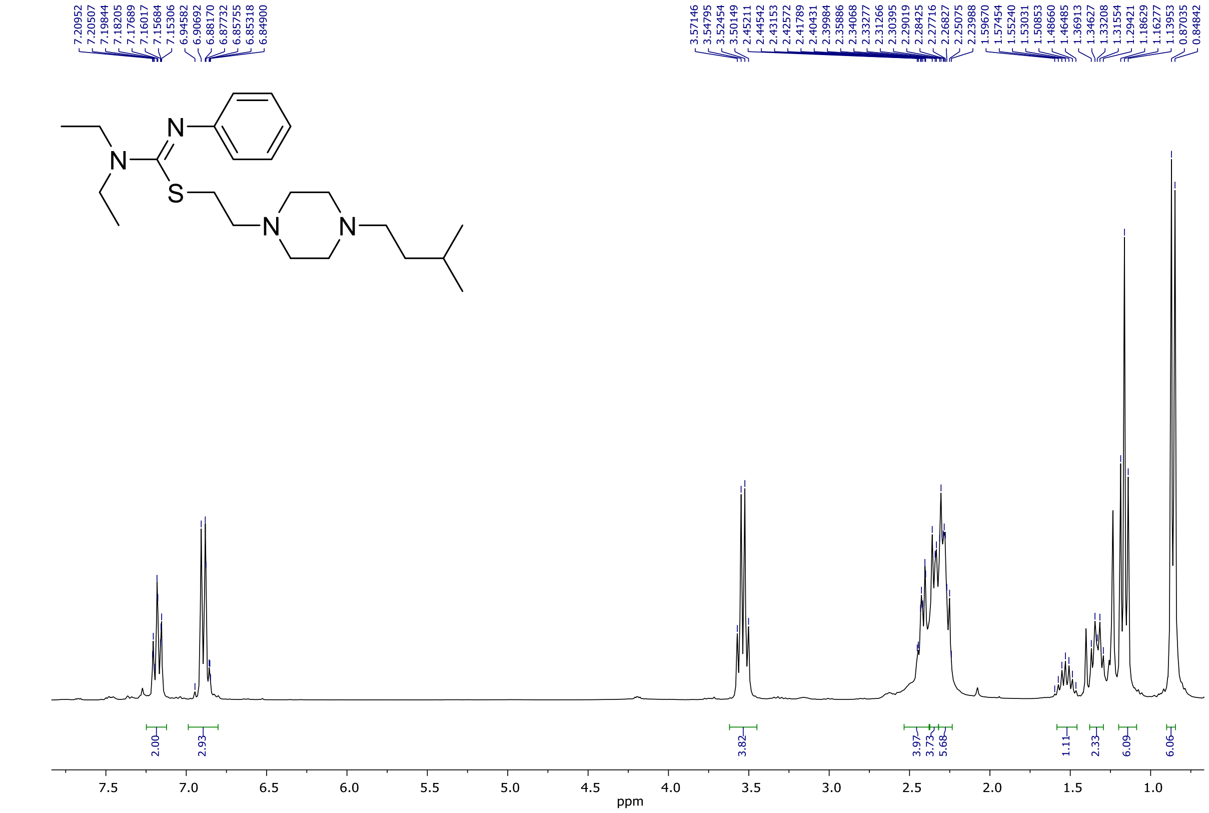

^{1}H NMR (300 MHz, $CDCl_3$); *2-(4-Isopentylpiperazin-1-yl))ethyl (Z)-N,N-diethyl-N'-phenylcarbamimidothioate* **3c**

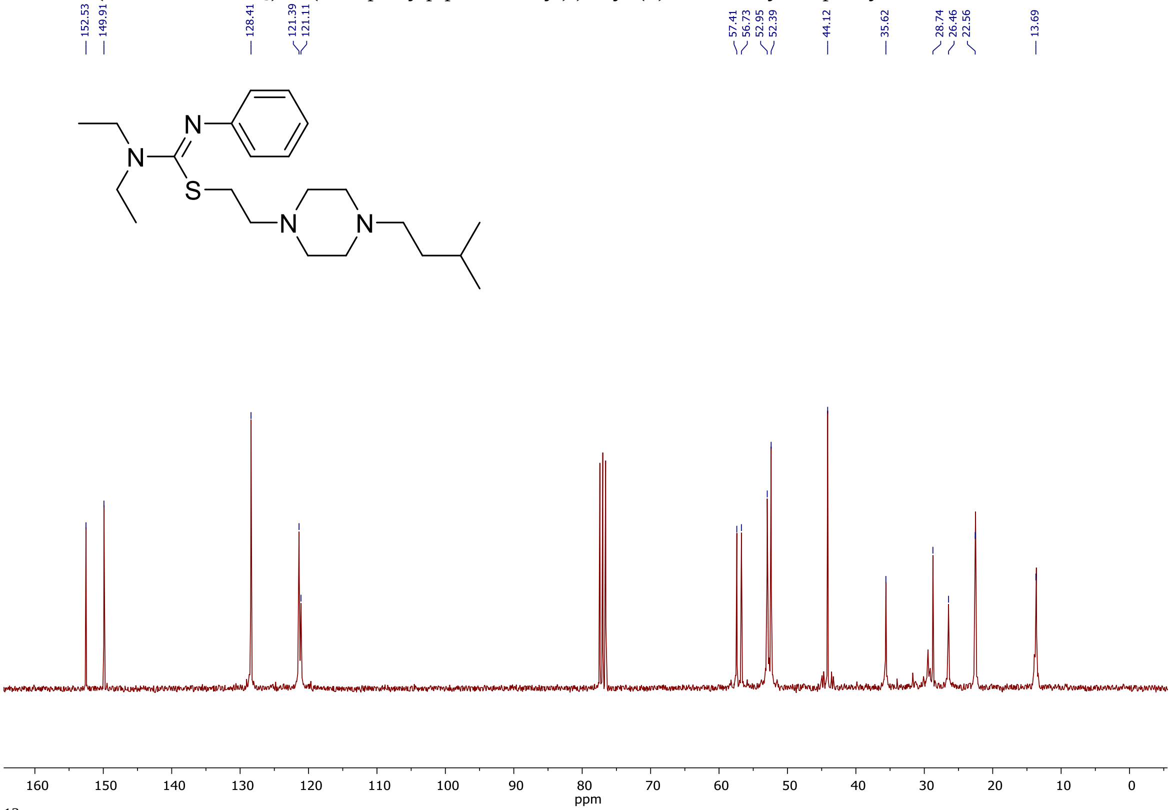

^{13}C NMR (75 MHz, $CDCl_3$); *2-(4-Isopentylpiperazin-1-yl))ethyl (Z)-N,N-diethyl-N'-phenylcarbamimidothioate* **3c**

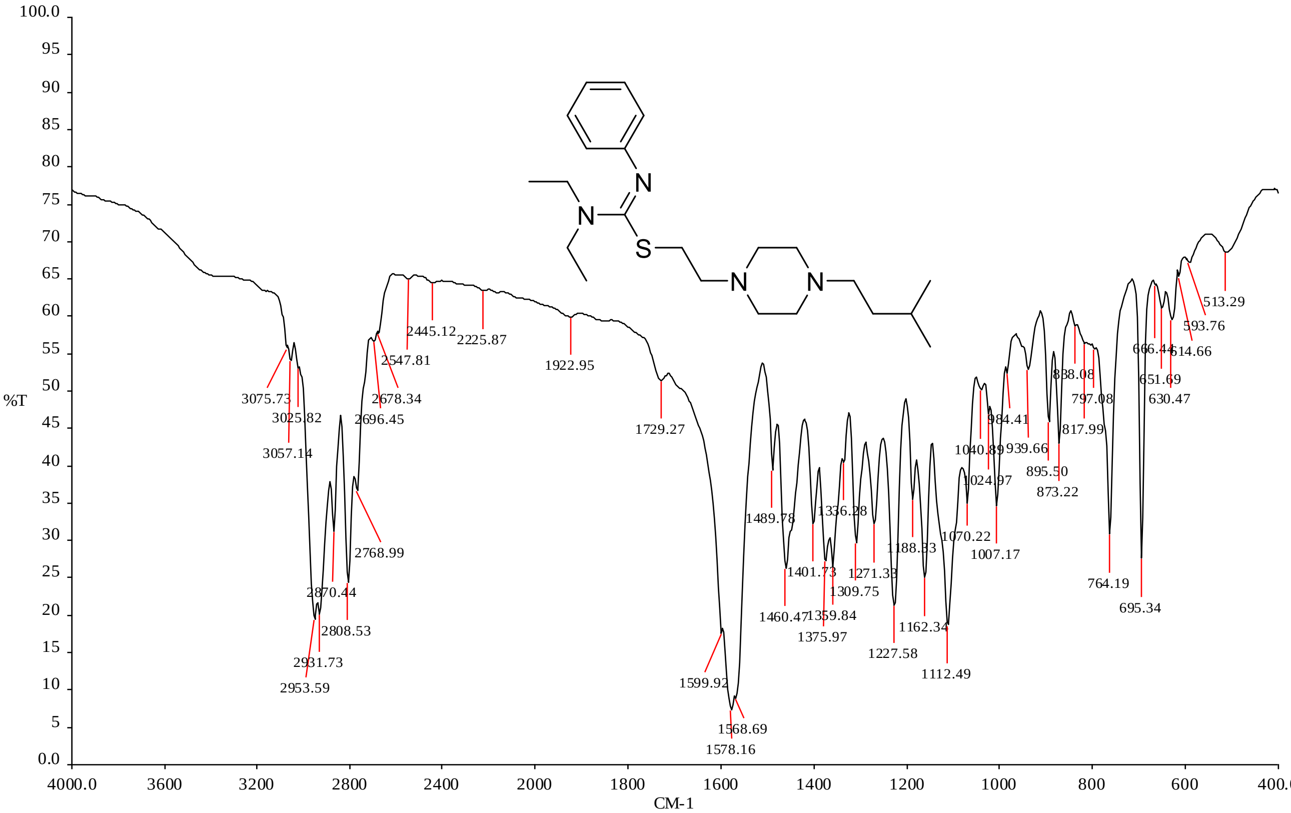

Mr. Moradi **Sample: D 2**

Acquired : 3 Jan 2007 6:58 using AcqMethod f1.M
Instrument : MSD
Sample Name: D2
Misc Info :
Vial Number: 1

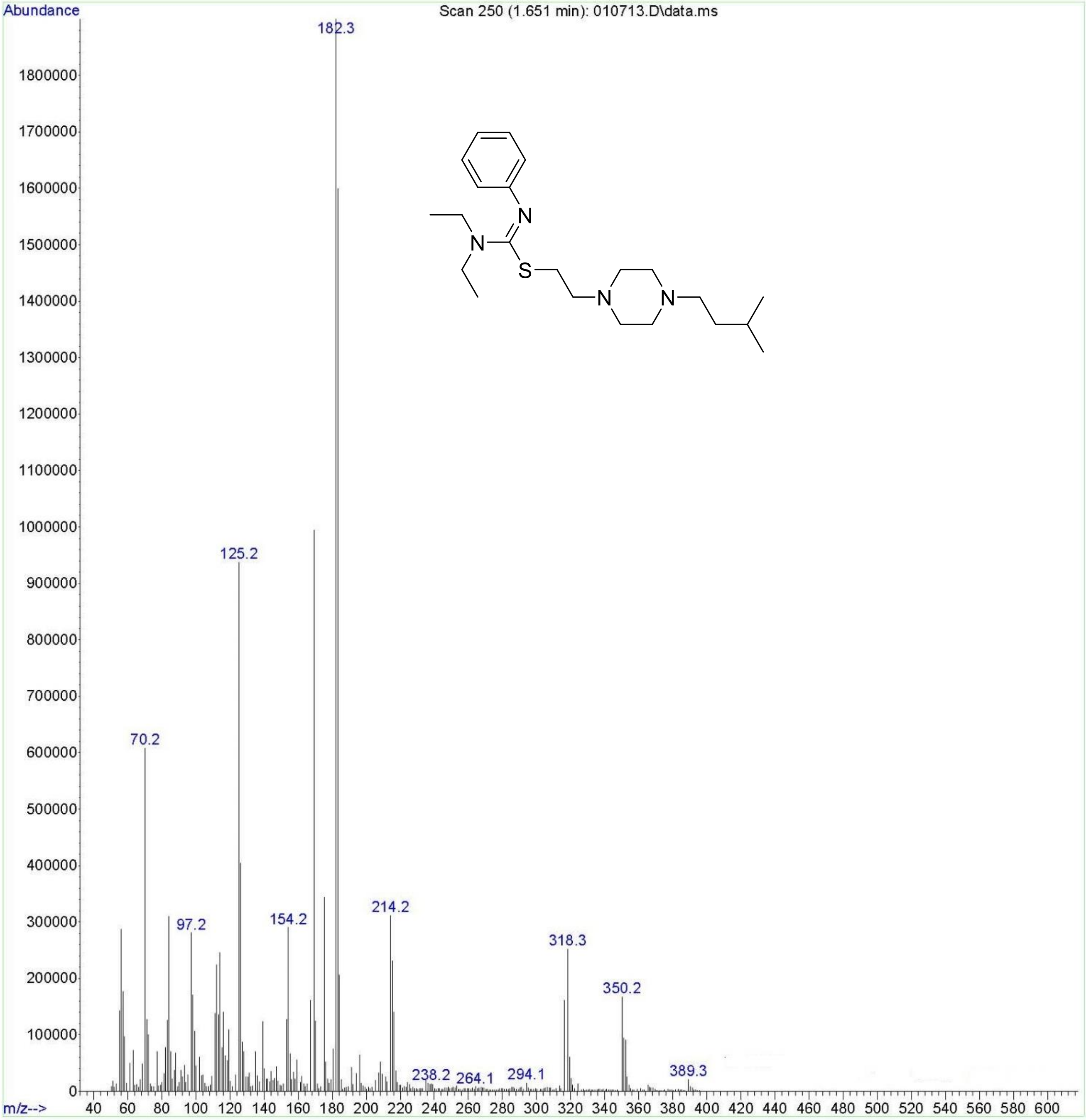

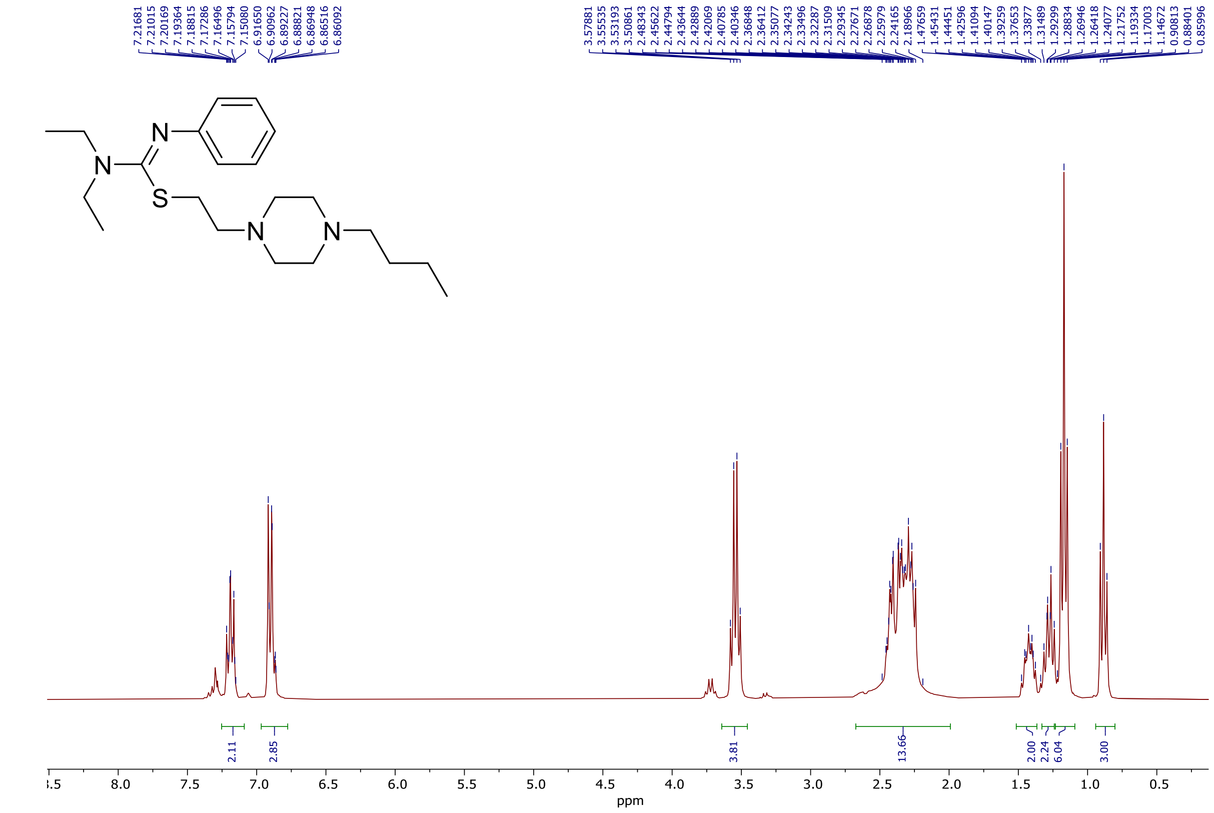

^{1}H NMR (300 MHz, $CDCl_3$); *2-(4-Butylpiperazin-1-yl))ethyl (Z)-N,N-diethyl-N'-phenylcarbamimidothioate* **3d**

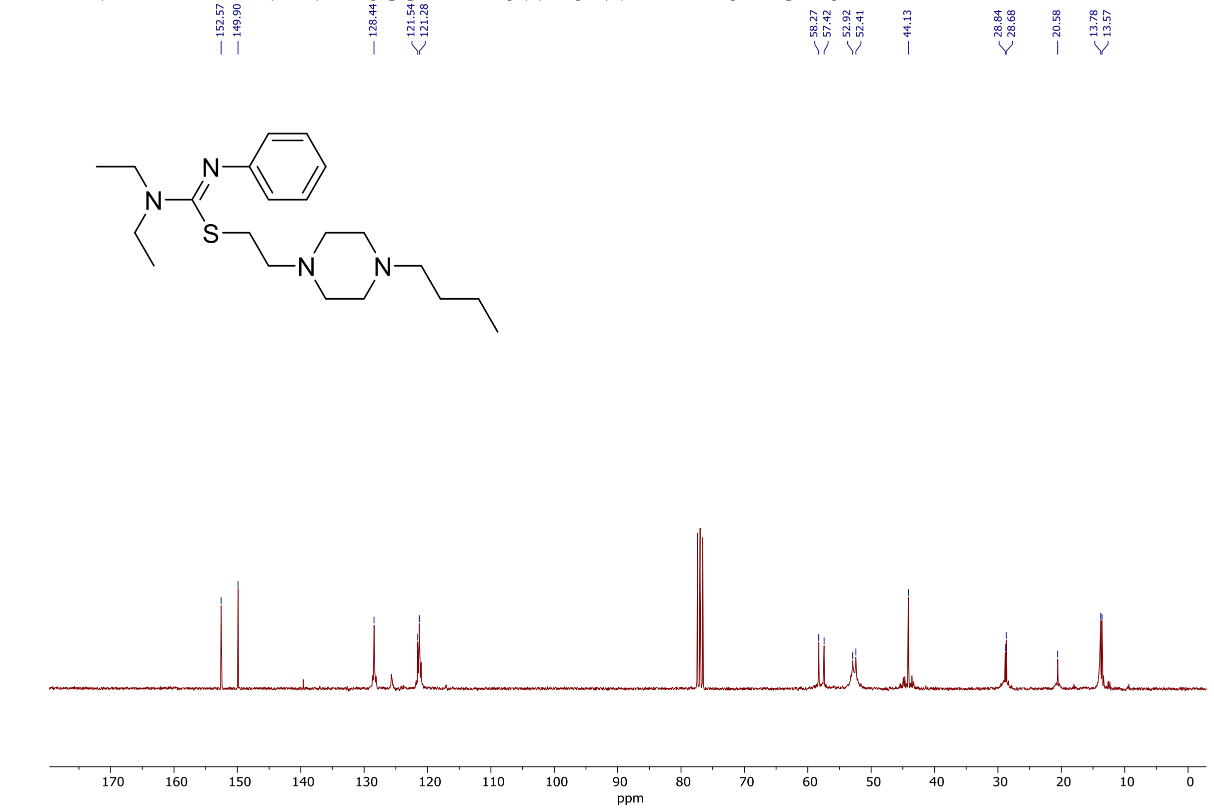

^{13}C NMR (75 MHz, $CDCl_3$); *2-(4-Butylpiperazin-1-yl))ethyl (Z)-N,N-diethyl-N'-phenylcarbamimidothioate* **3d**

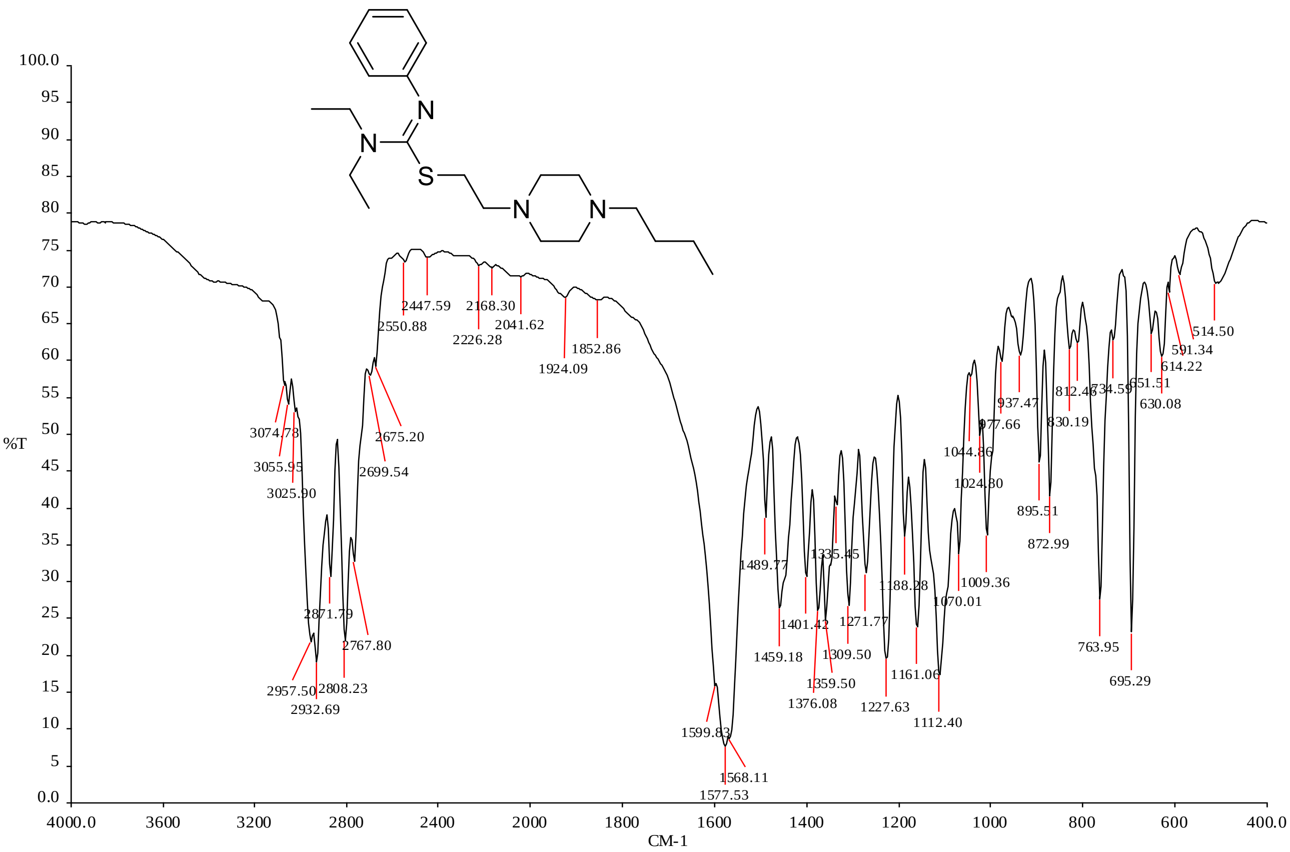

Mr. Moradi **Sample: D 14**

Acquired : 3 Jan 2007 7:34 using AcqMethod f1.M
Instrument : MSD
Sample Name: D14
Misc Info :
Vial Number: 1

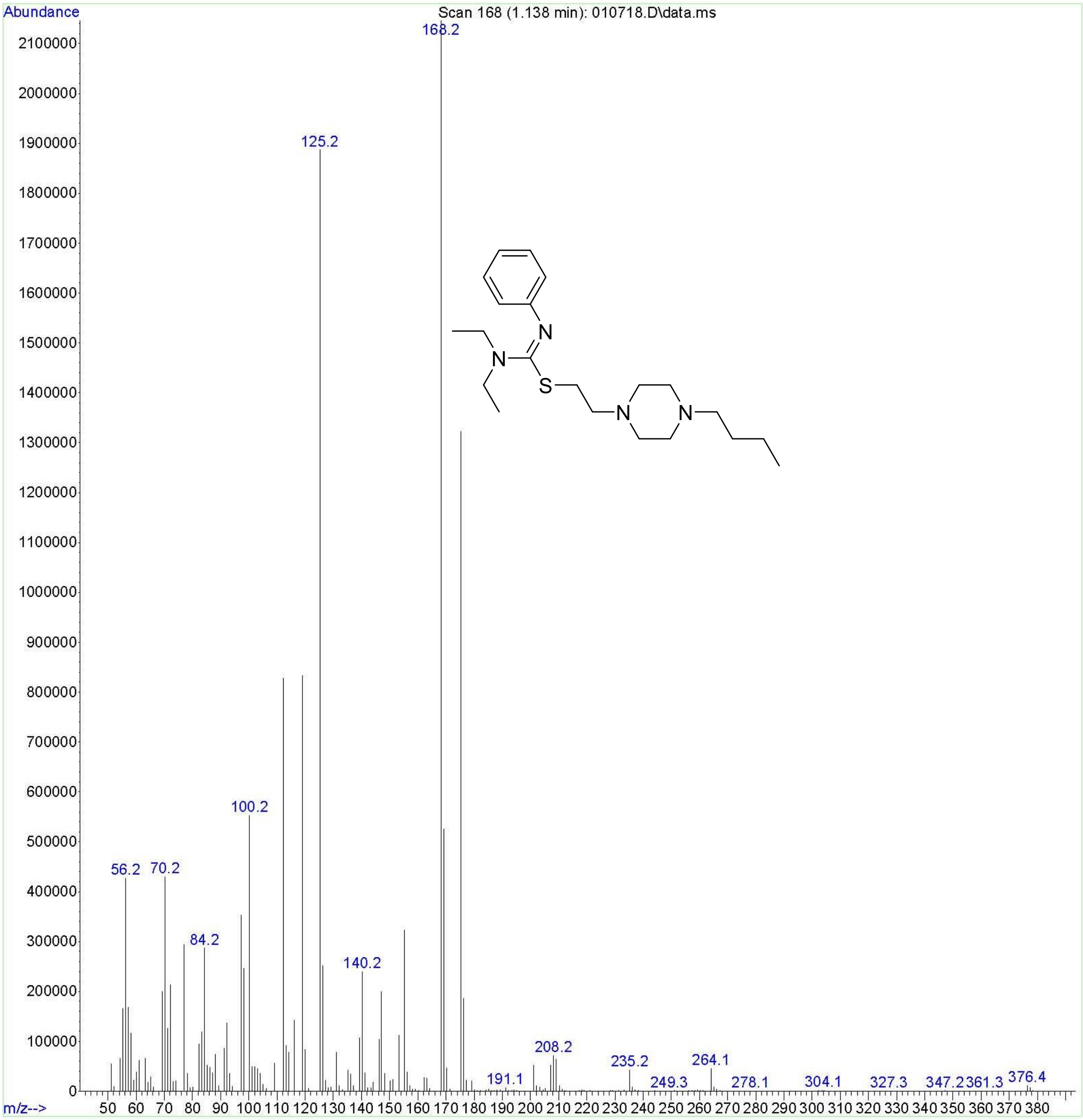

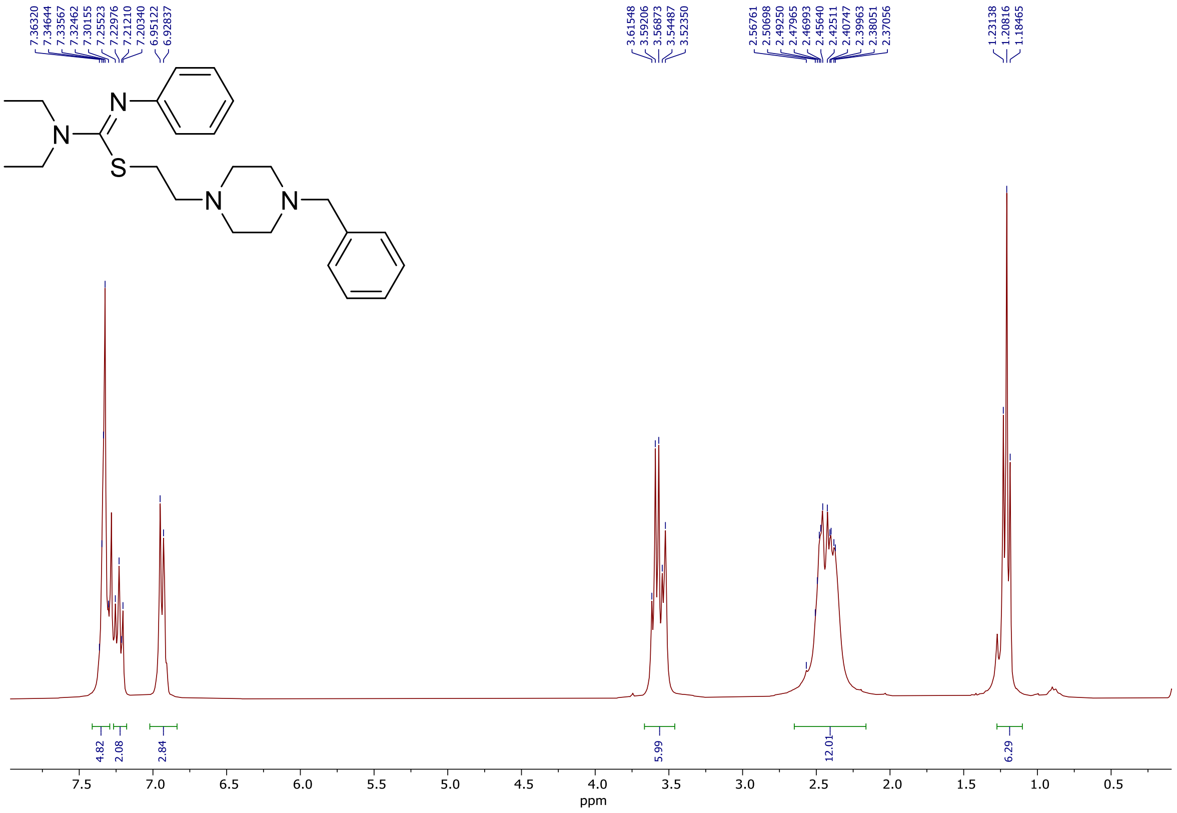

^{1}H NMR (300 MHz, $CDCl_3$); *2-(4-Benzylpiperazin-1-yl))ethyl (Z)-N,N-diethyl-N'-phenylcarbamimidothioate* **3e**

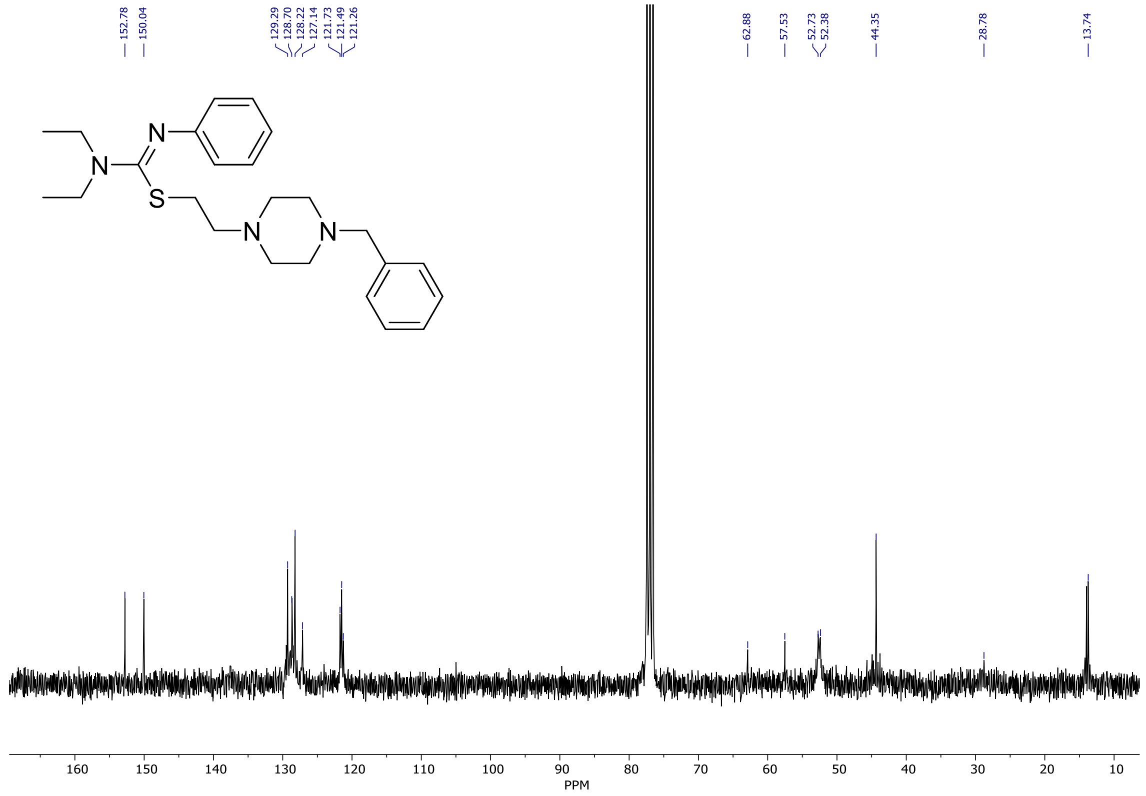

^{13}C NMR (75 MHz, $CDCl_3$); *2-(4-Benzylpiperazin-1-yl))ethyl (Z)-N,N-diethyl-N'-phenylcarbamimidothioate* **3e**

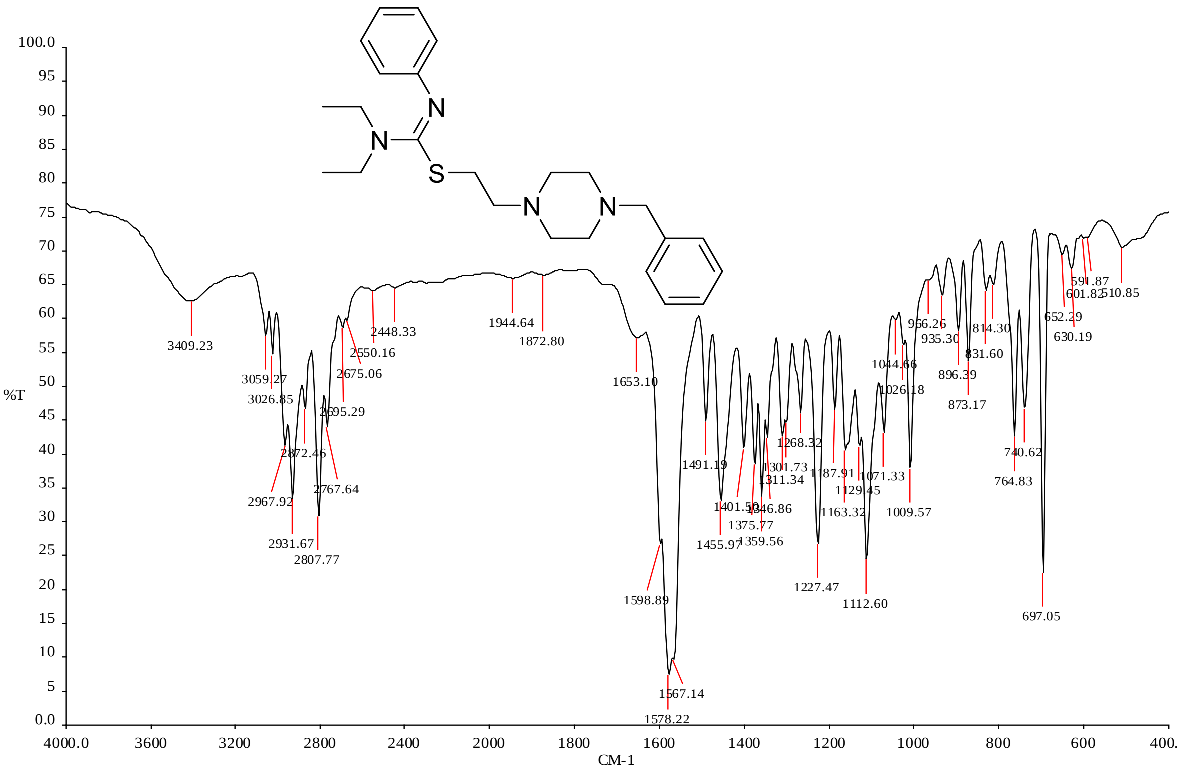

Mr. Moradi **Sample: D 10**

[illegible]
Acquired : 3 Jan 2007 6:43 using AcqMethod f1.M
Instrument : MSD
Sample Name: D10
Misc Info :
Vial Number: 1

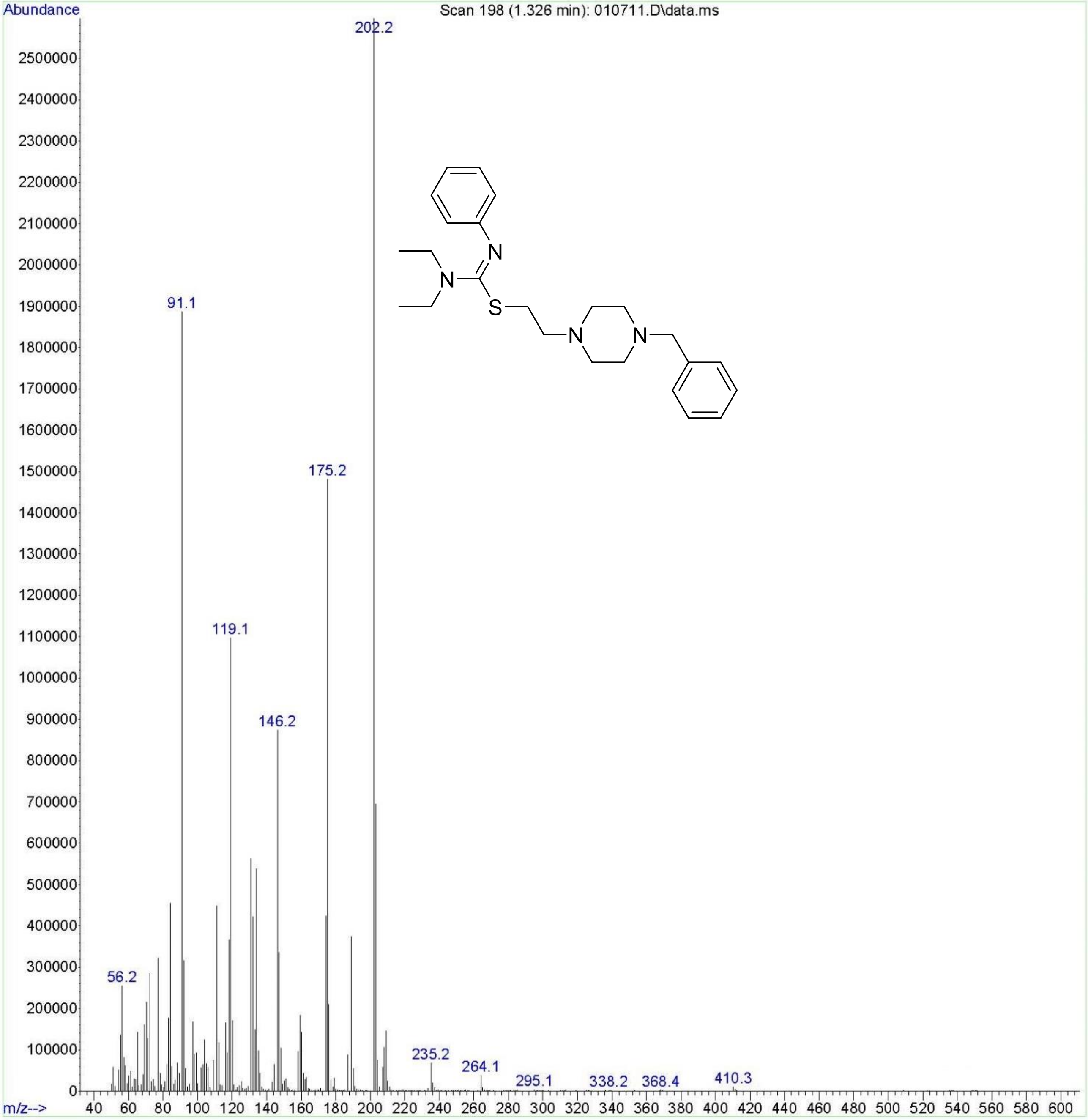

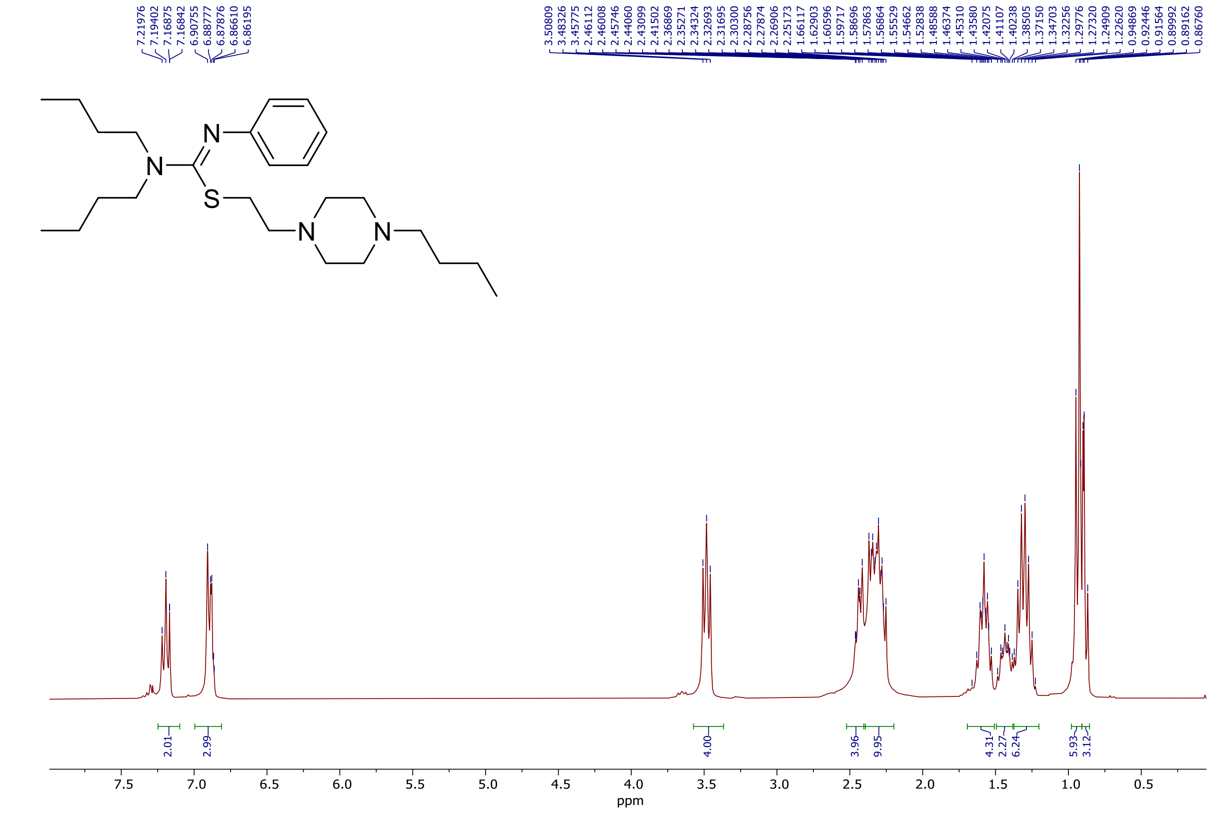

^{1}H NMR (75 MHz, $CDCl_3$); *2-(4-Butylpiperazin-1-yl)ethyl (Z)-N,N-dibutyl-N'-phenylcarbamimidothioate* **3f**

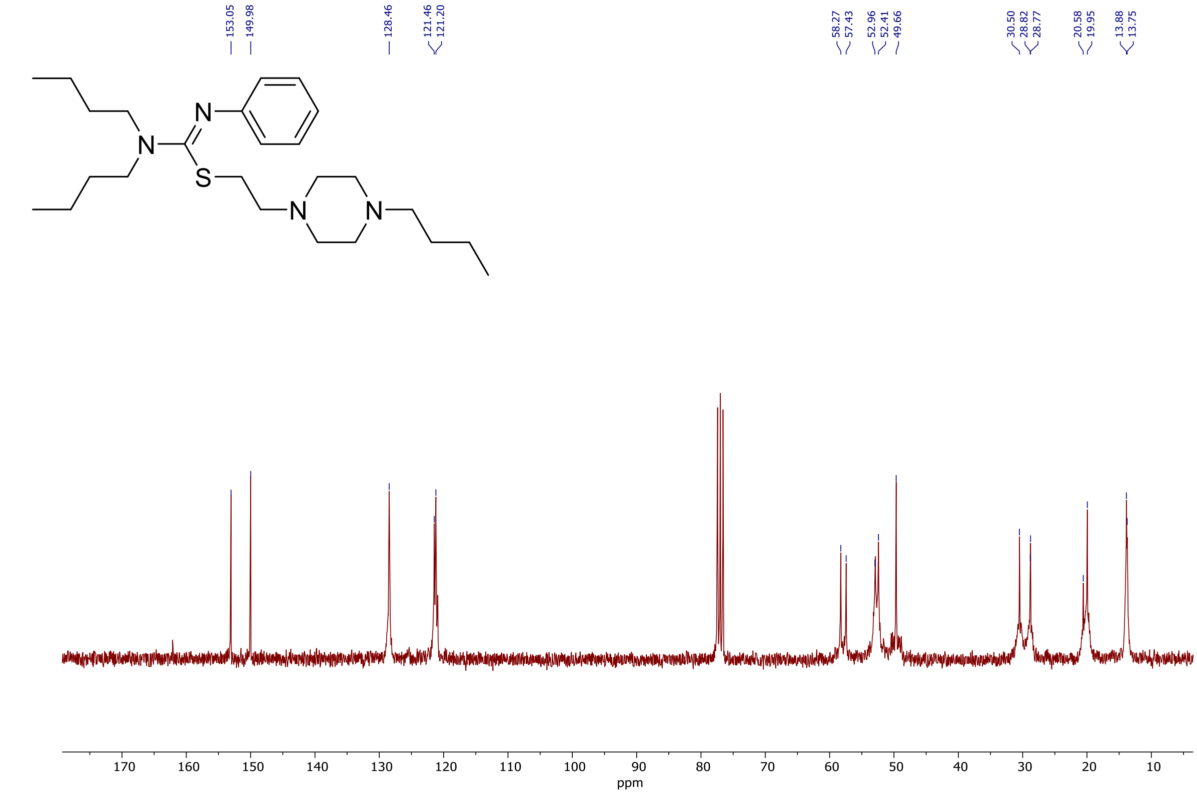

^{13}C NMR (75 MHz, $CDCl_3$); *2-(4-Butylpiperazin-1-yl)ethyl (Z)-N,N-dibutyl-N'-phenylcarbamimidothioate* **3f**

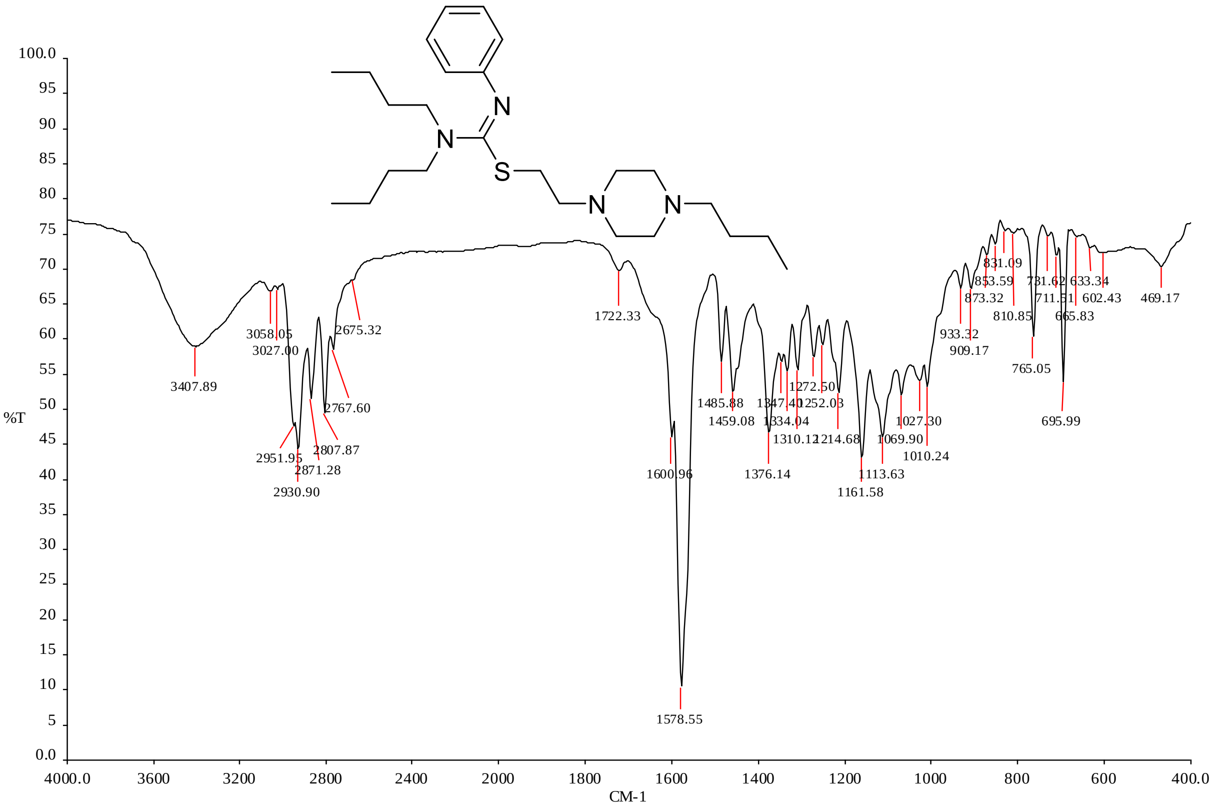

Mr. Moradi **Sample: D 16**

Acquired : 3 Jan 2007 6:51 using AcqMethod f1.M
Instrument : MSD
Sample Name: D16
Misc Info :
Vial Number: 1

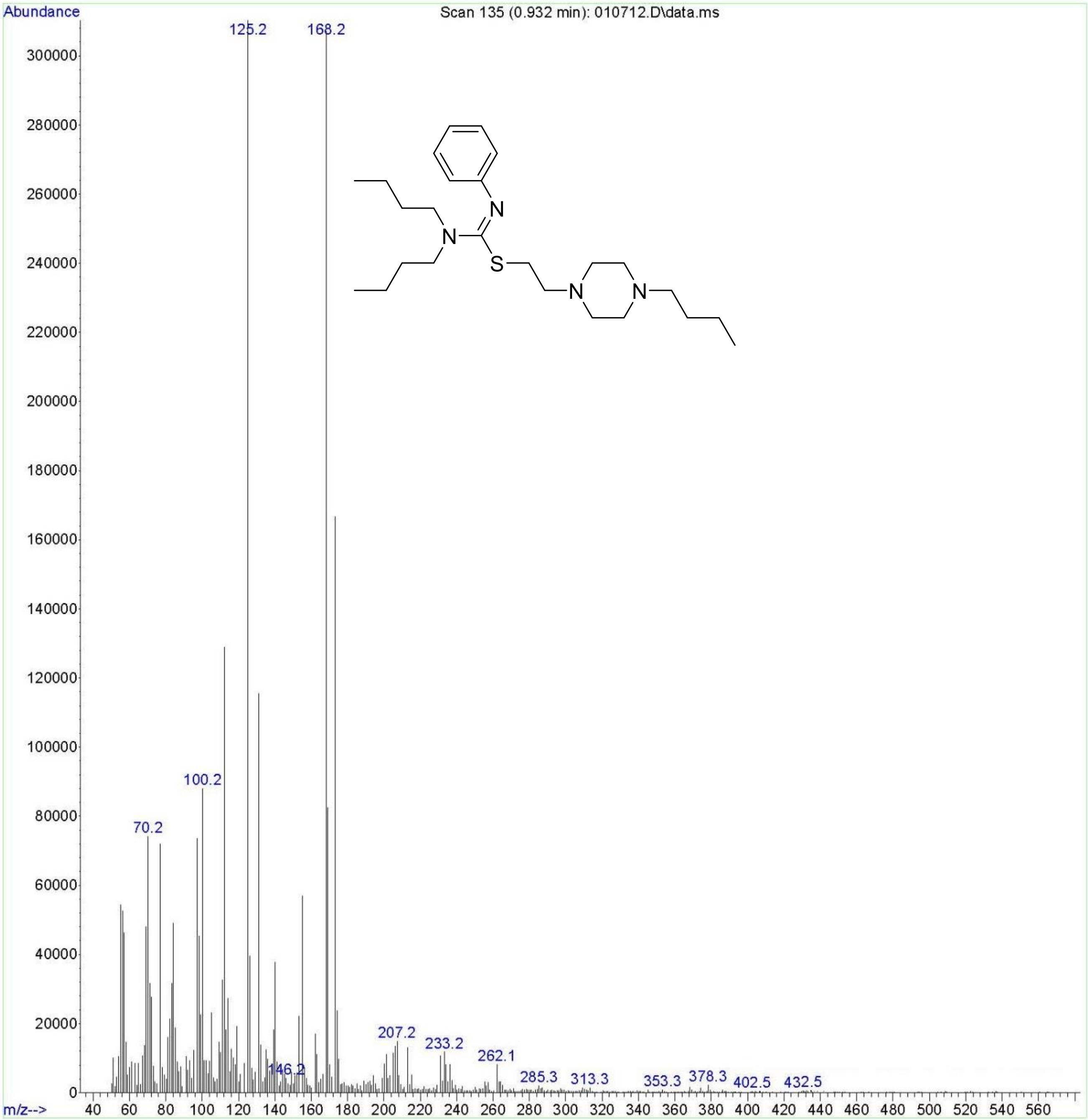

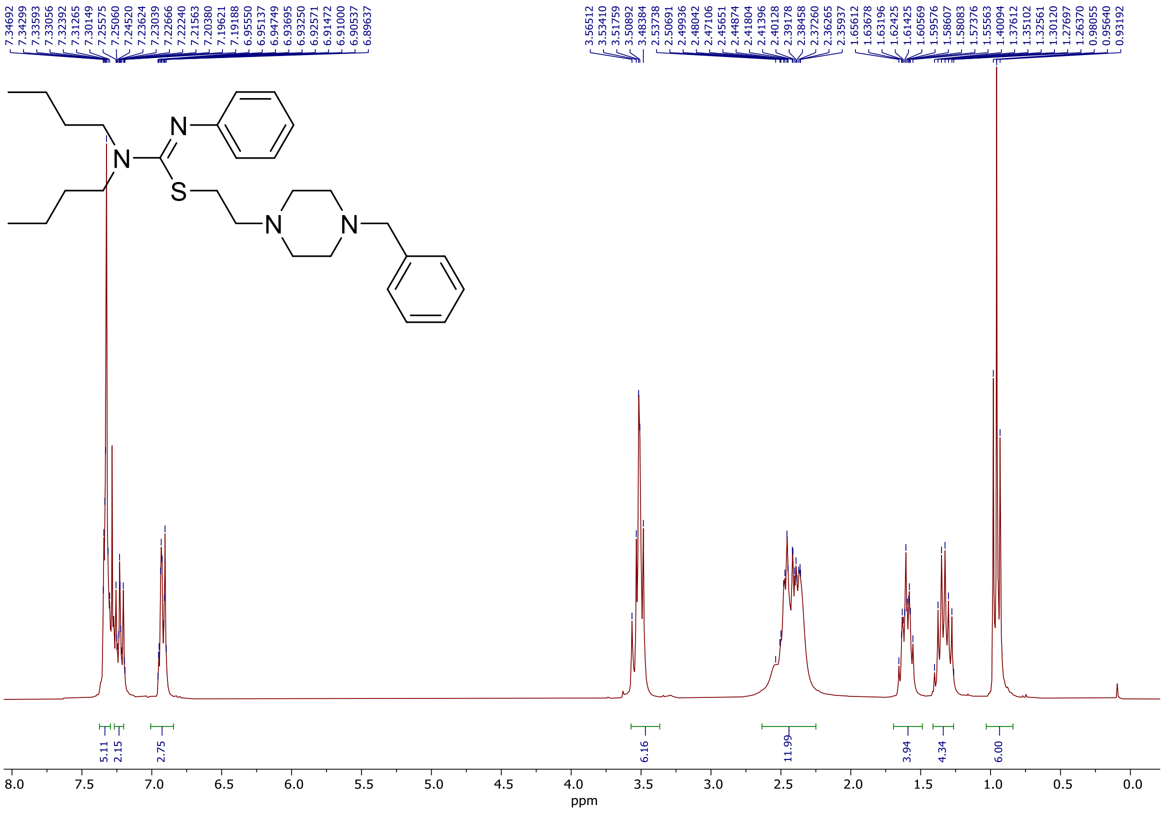

^{1}H NMR (300 MHz, $CDCl_3$); *2-(4-Benzylpiperazin-1-yl)ethyl (Z)-N,N-dibutyl-N'-phenylcarbamimidothioate* **3g**

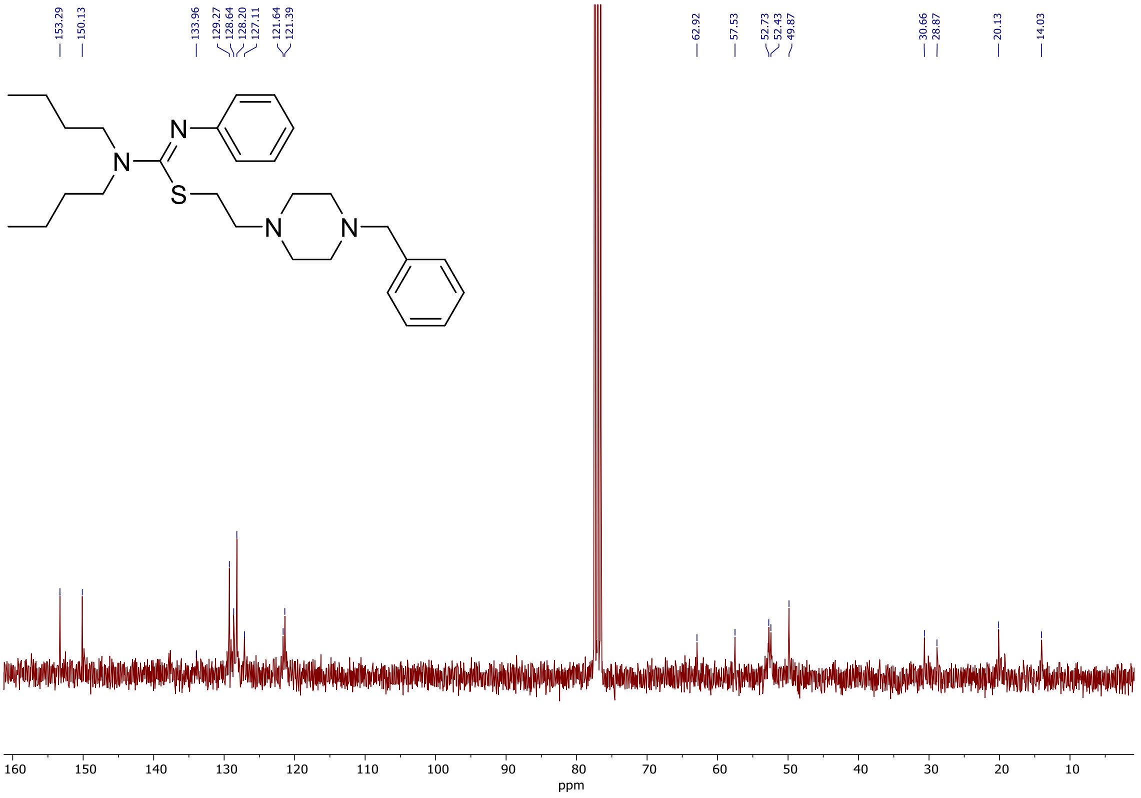

^{13}C NMR (75 MHz, $CDCl_3$); *2-(4-Benzylpiperazin-1-yl)ethyl (Z)-N,N-dibutyl-N'-phenylcarbamimidothioate* **3g**

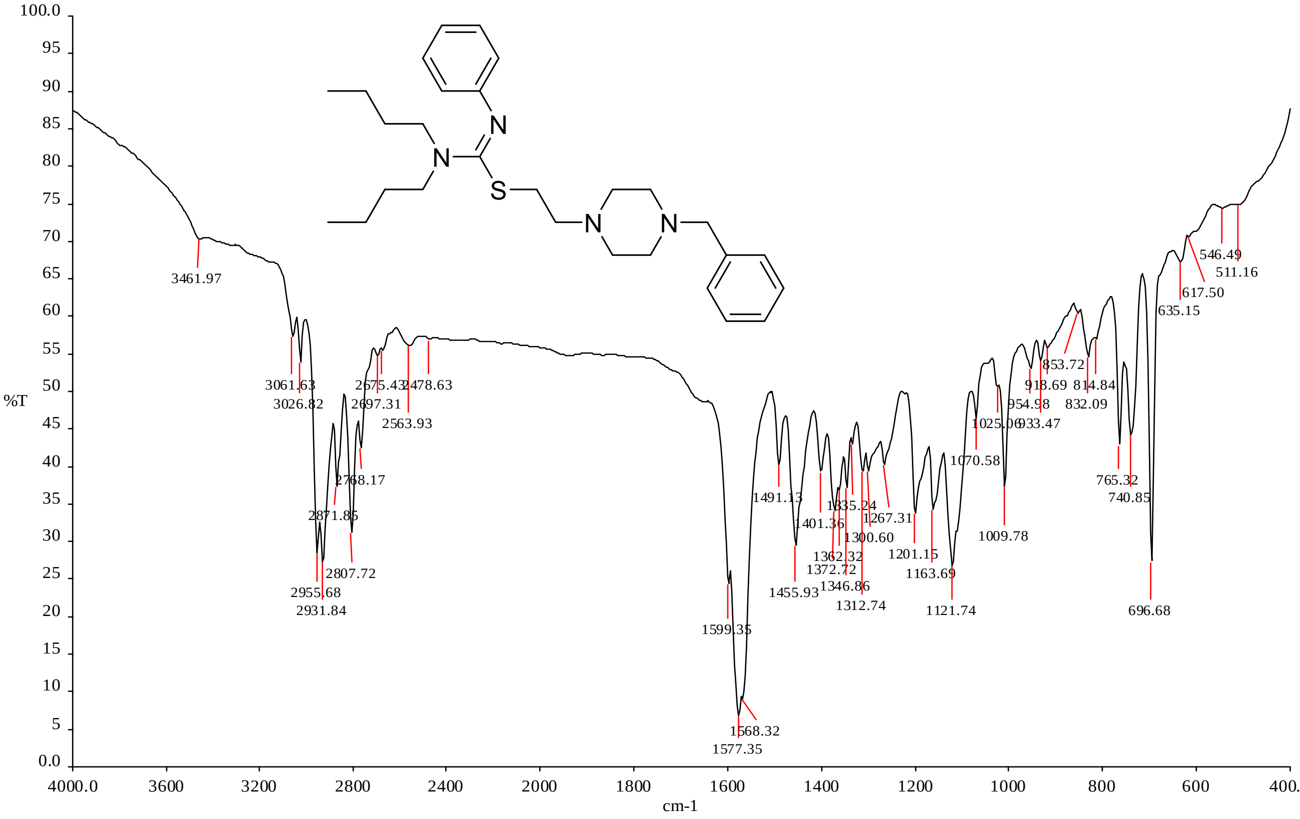

Mr. Moradi **Sample: D 13**

Acquired : 3 Jan 2007 7:27 using AcqMethod f1.M
Instrument : MSD
Sample Name: D13
Misc Info :
Vial Number: 1

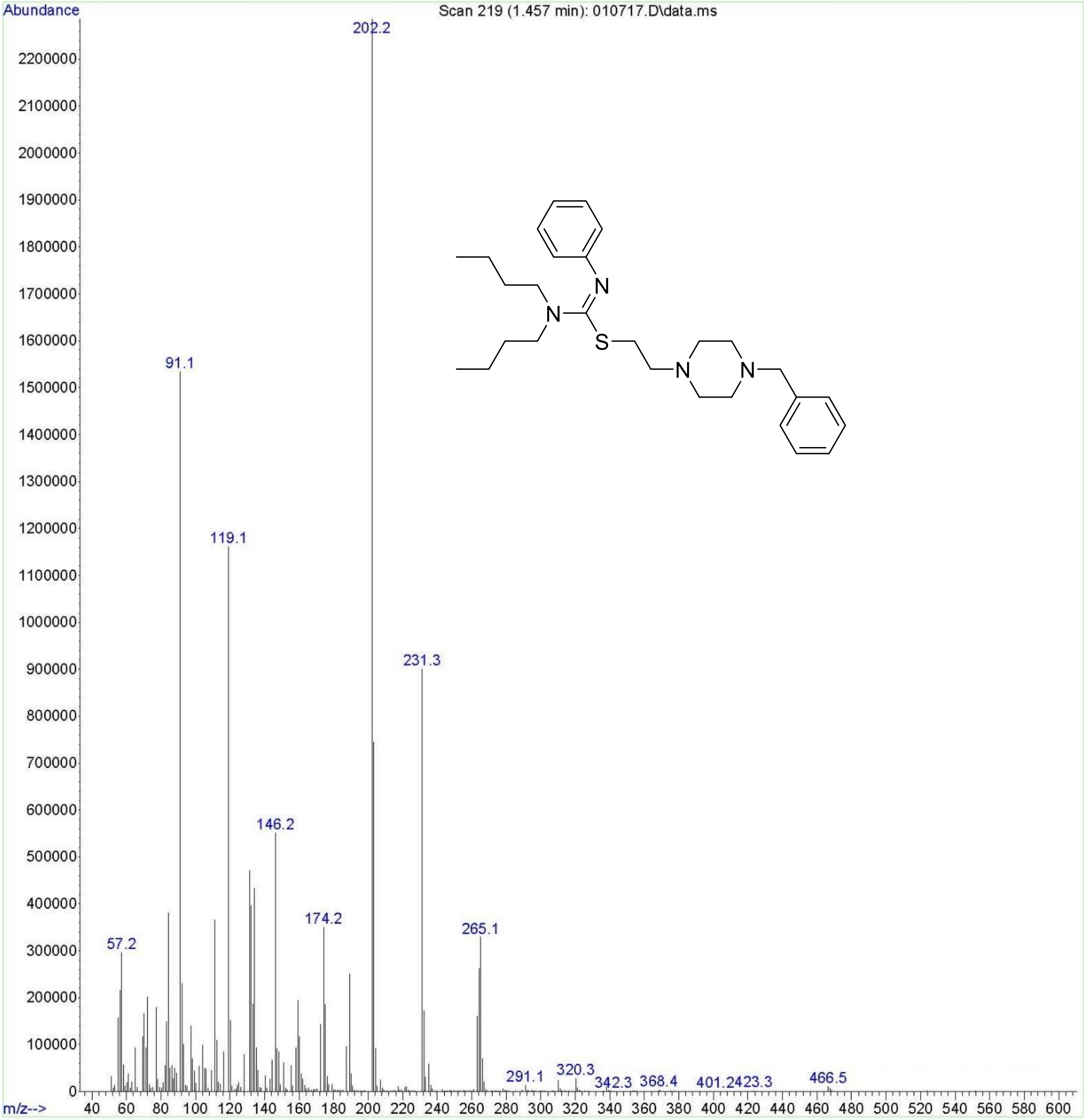

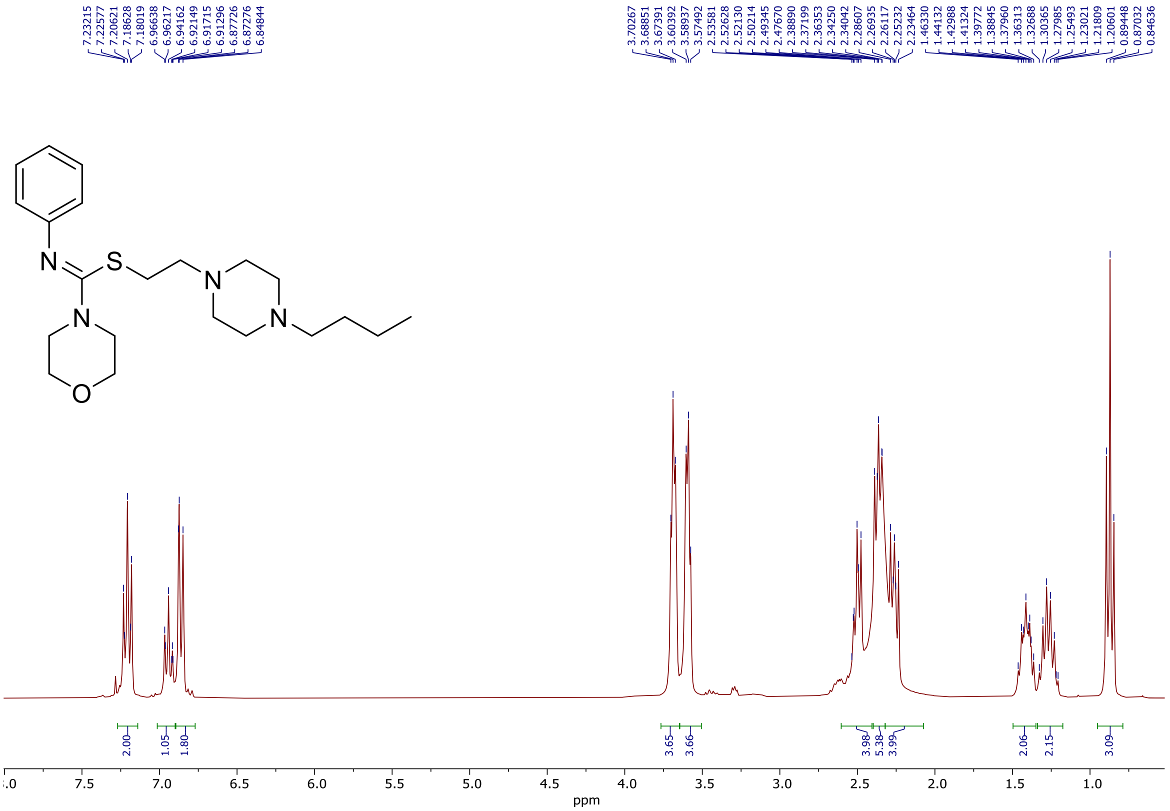

^{1}H NMR (300 MHz, $CDCl_3$); *2-(4-Butylpiperazin-1-yl)ethyl (Z)-N-phenylmorpholine-4-carbimidothioate* **3h**

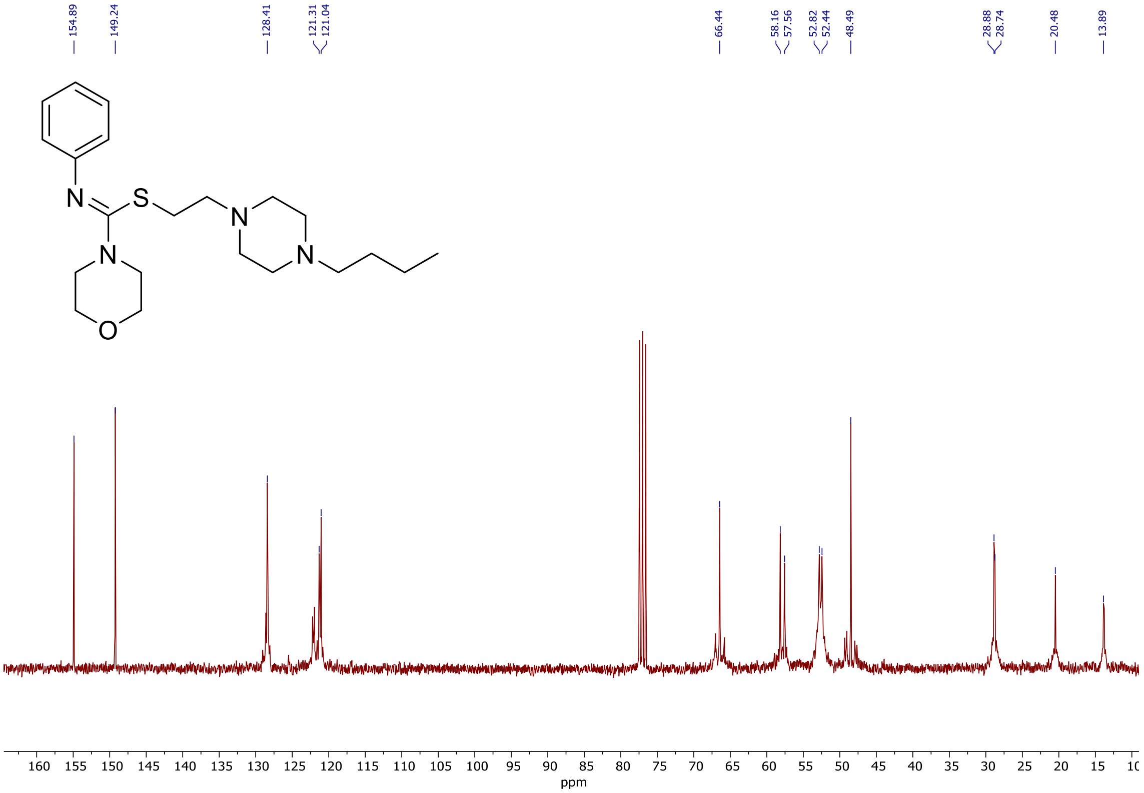

^{13}C NMR (75 MHz, $CDCl_3$); *2-(4-Butylpiperazin-1-yl)ethyl (Z)-N-phenylmorpholine-4-carbimidothioate* **3h**

The Perkin-Elmer Spectrum RXI FT-IR Spectrometer *Date: 5/30/2022* *(401/3/9)* *Time: 4:12:50 PM*

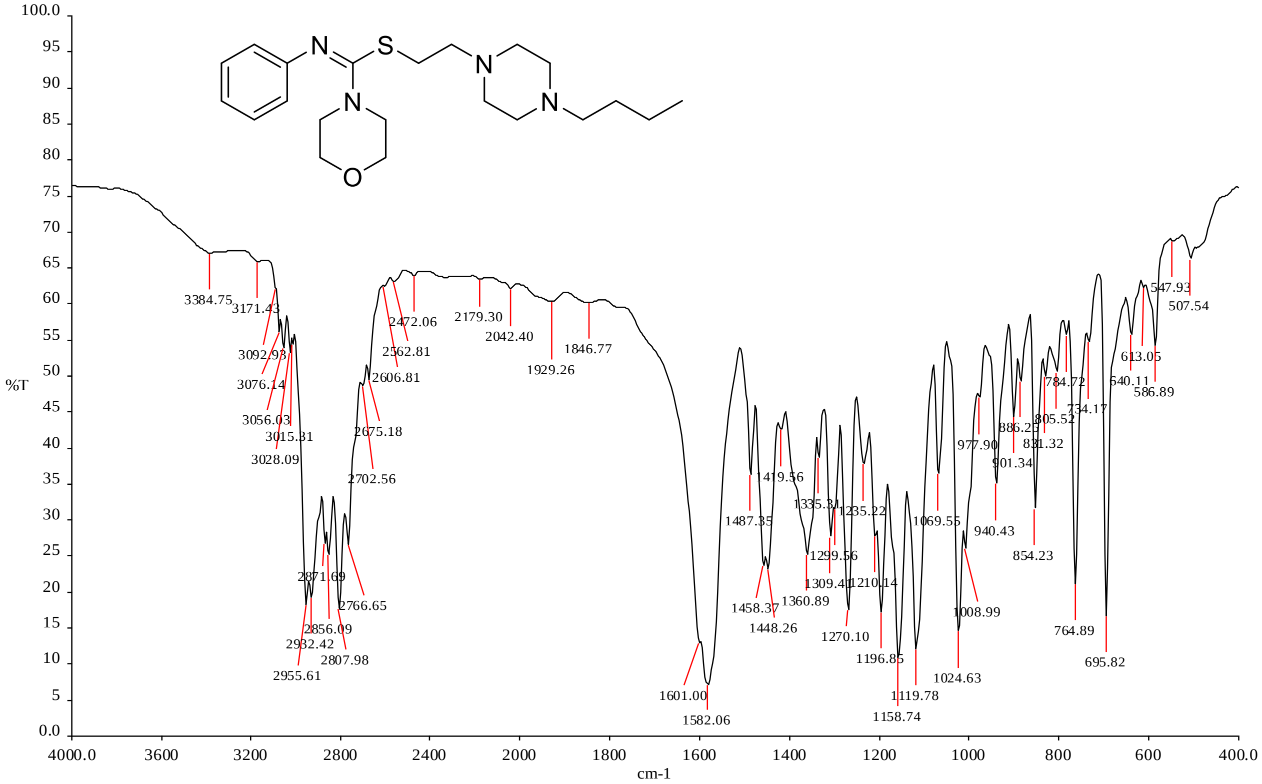

Mr. Moradi **Sample: D 15**

Acquired : 3 Jan 2007 7:40 using AcqMethod f1.M
Instrument : MSD
Sample Name: D15
Misc Info :
Vial Number: 1

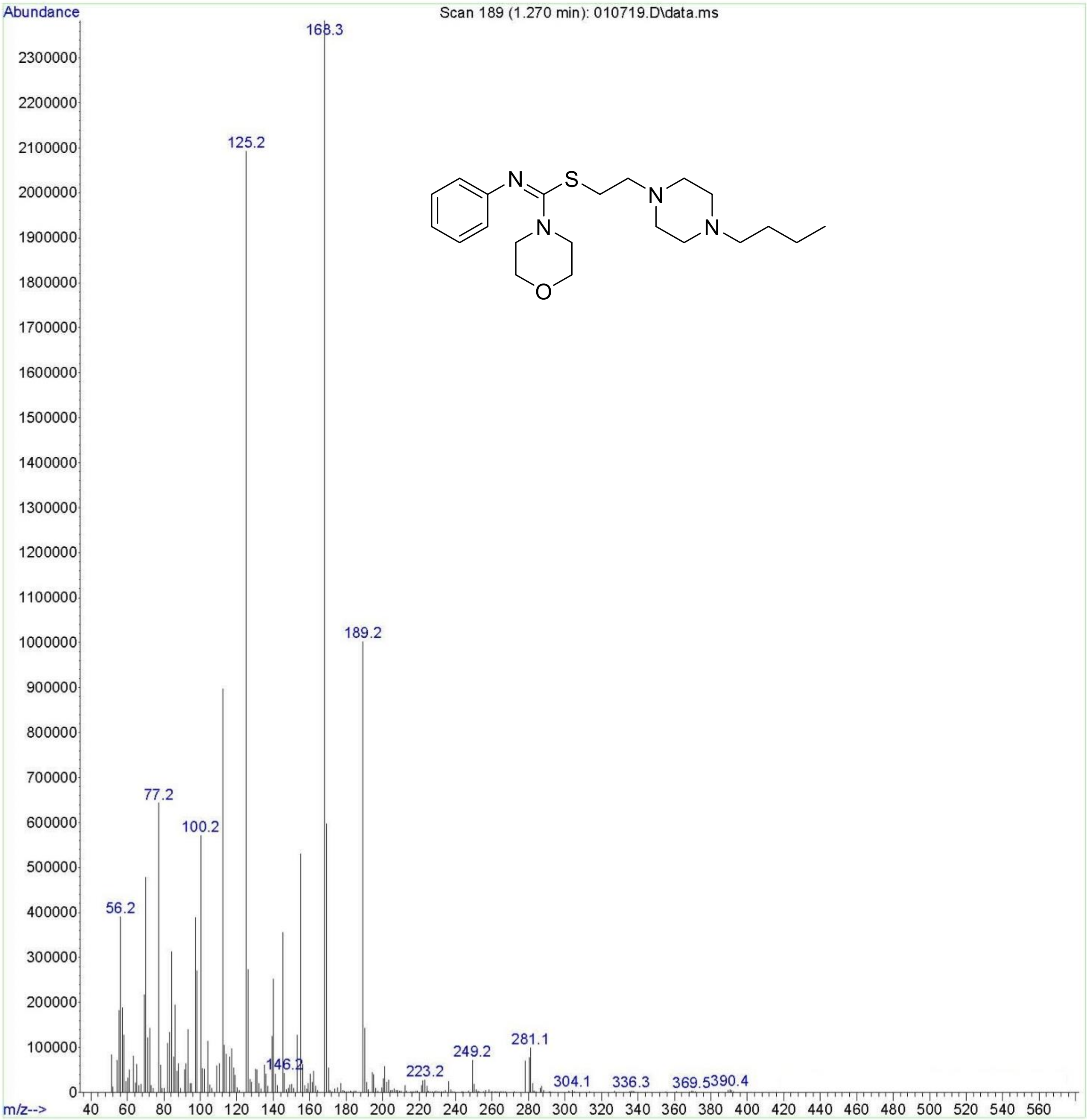

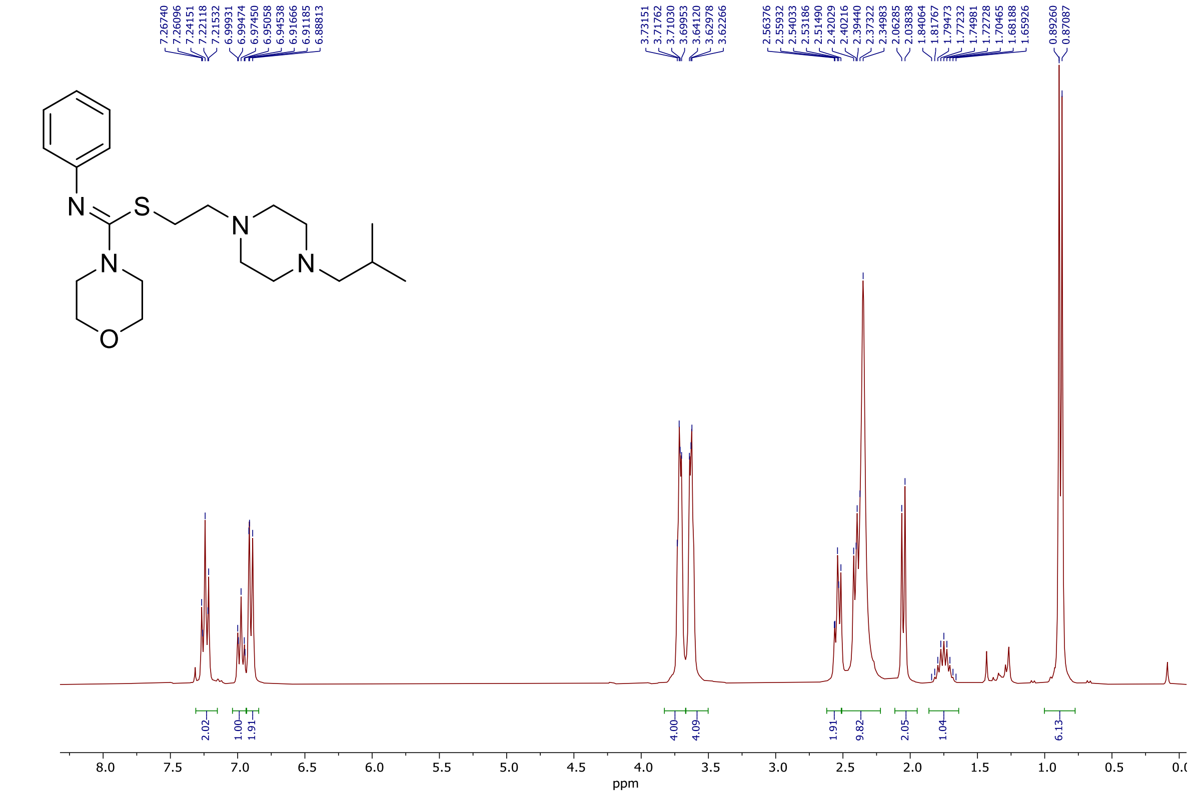

^{1}H NMR (300 MHz, $CDCl_3$); *2-(4-Isobutylpiperazin-1-yl)ethyl (Z)-N-phenylmorpholine-4-carbimidothioate* **3i**

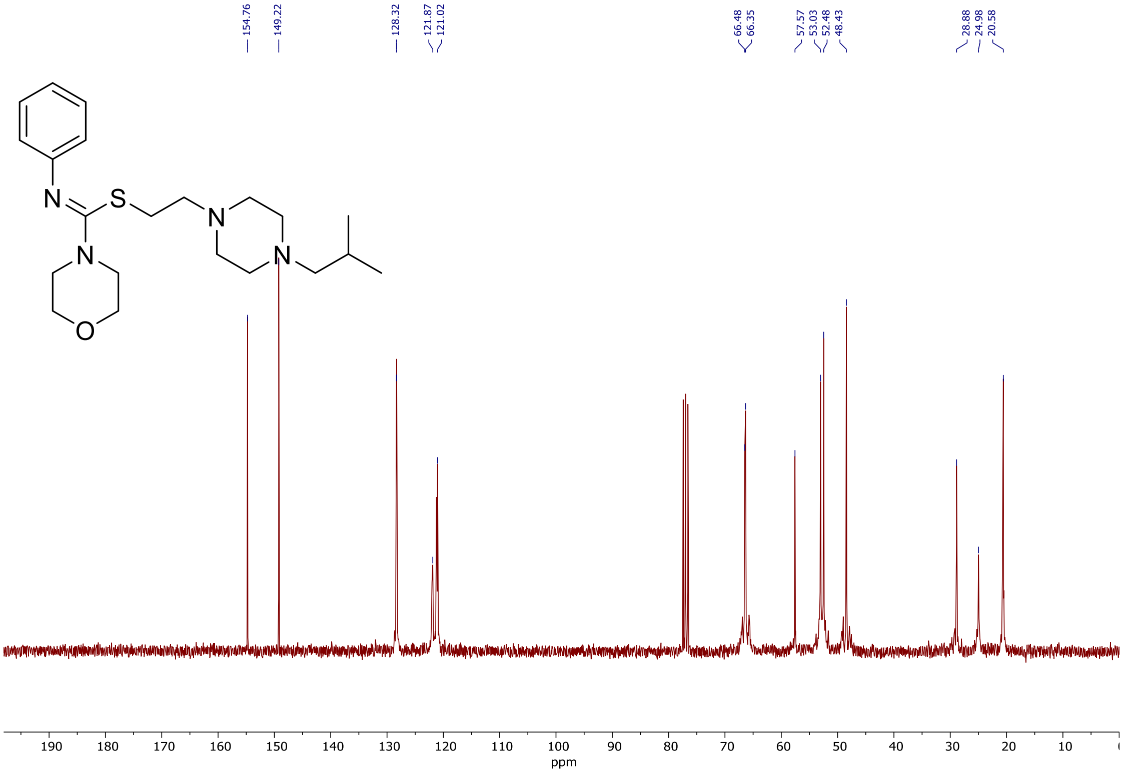

^{13}C NMR (75 MHz, $CDCl_3$); *2-(4-Isobutylpiperazin-1-yl)ethyl (Z)-N-phenylmorpholine-4-carbimidothioate* **3i**

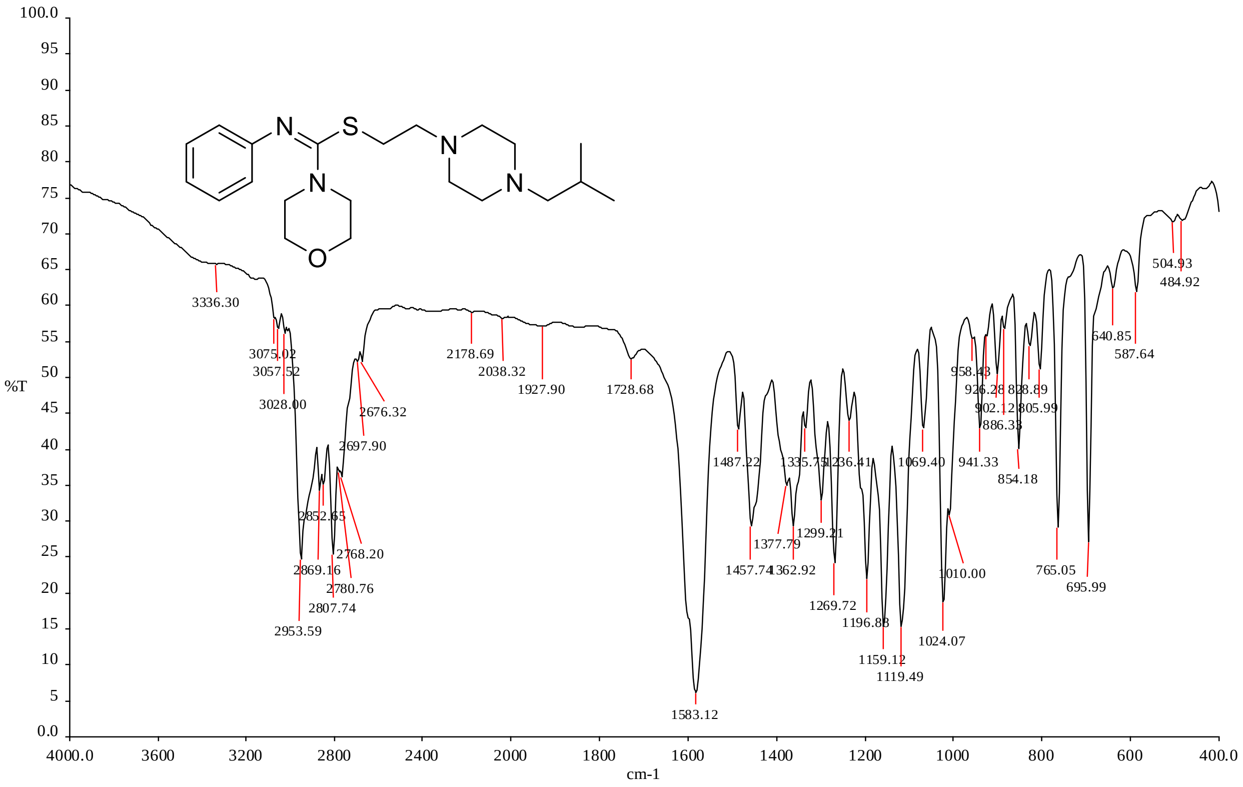

Mr. Moradi **Sample: D 4**

Acquired : 3 Jan 2007 7:13 using AcqMethod f1.M
Instrument : MSD
Sample Name: D4
Misc Info :
Vial Number: 1

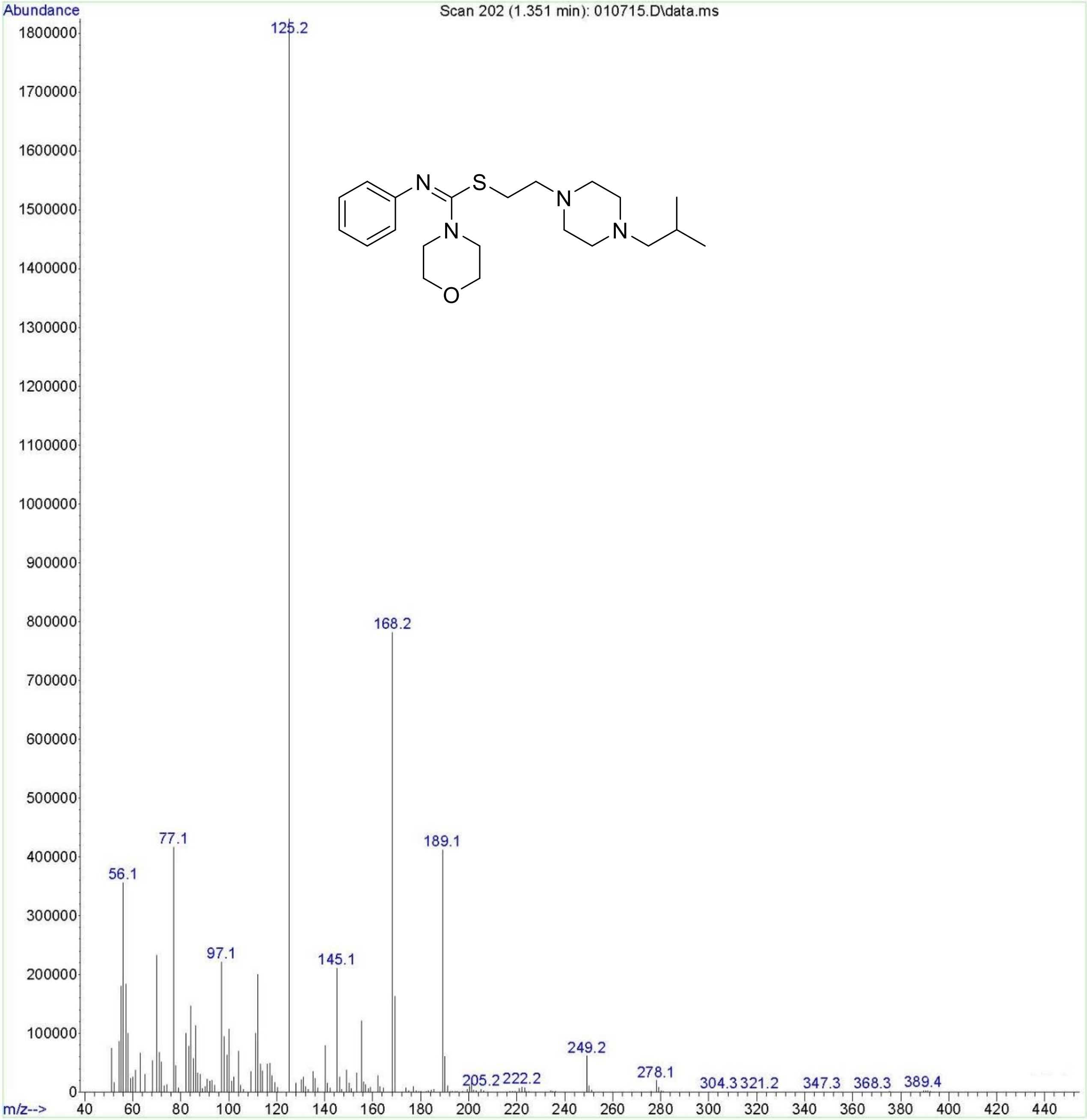

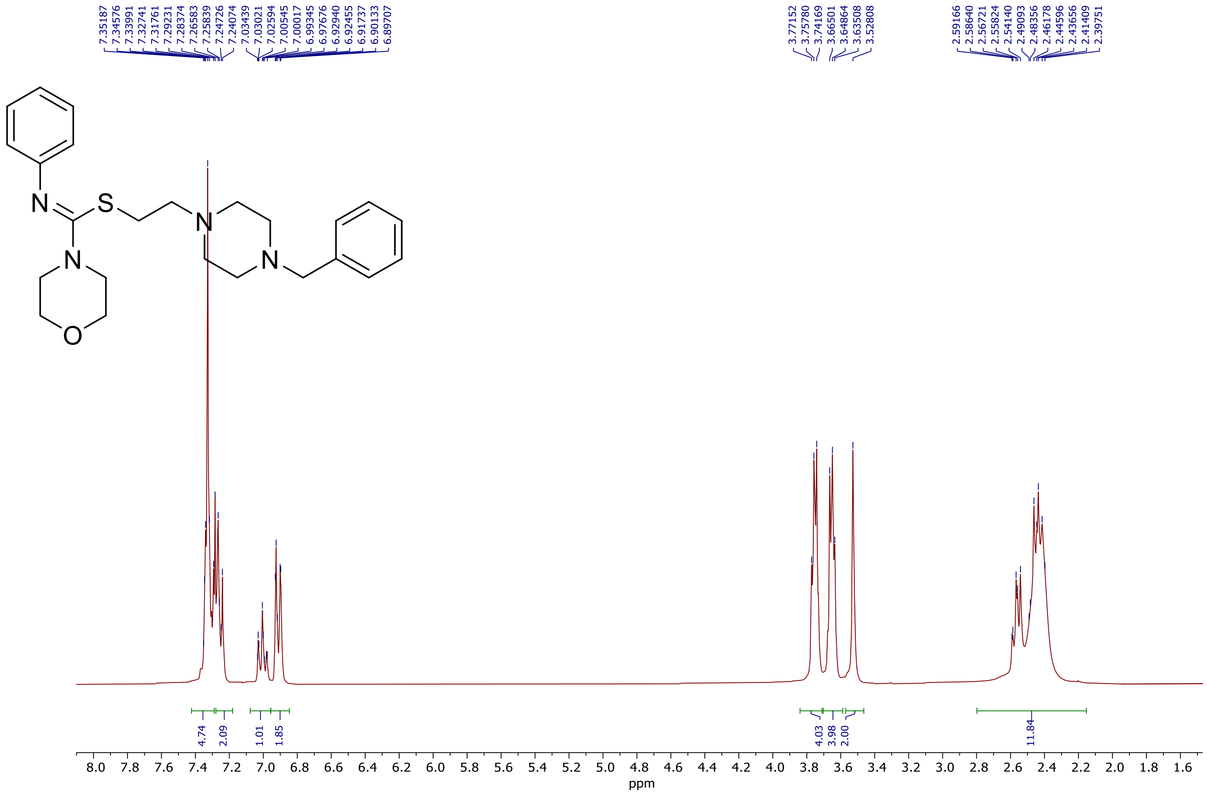

^{1}H NMR (75 MHz, $CDCl_3$); *2-(4-Benzylpiperazin-1-yl)ethyl (Z)-N-phenylmorpholine-4-carbimidothioate* **3j**

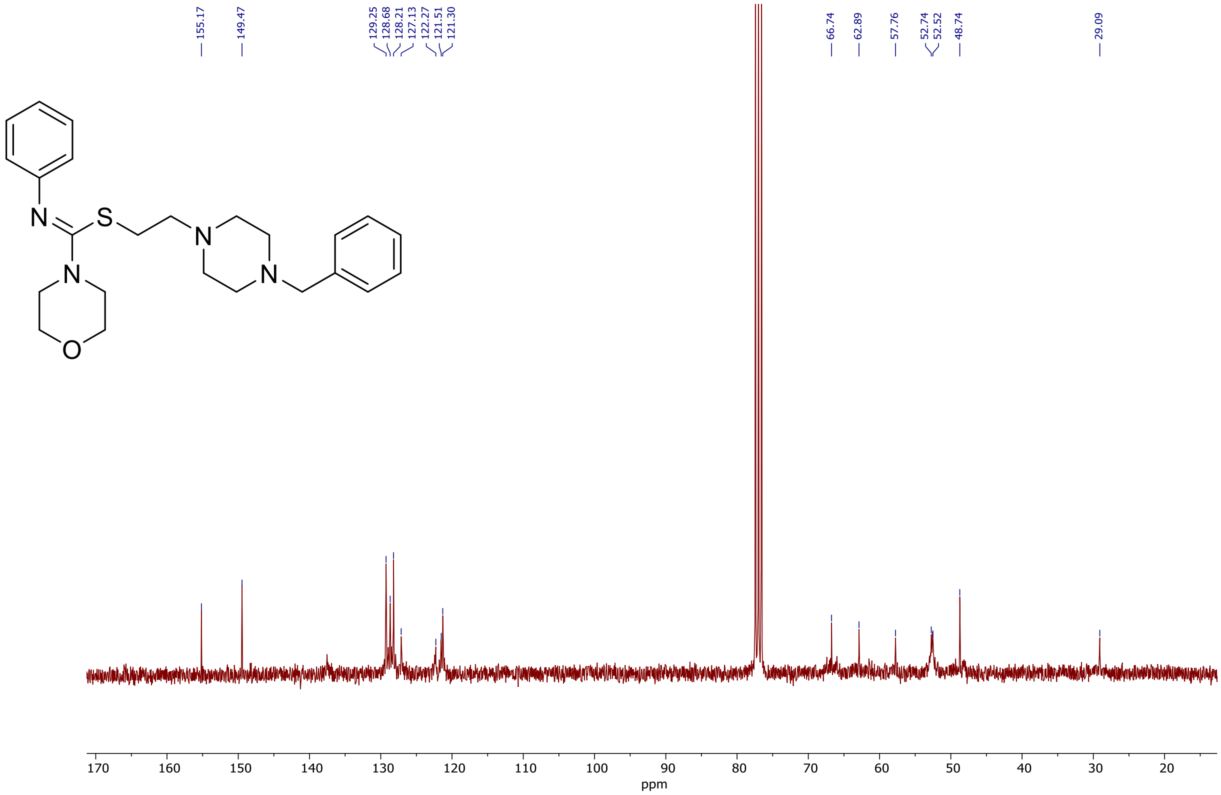

^{13}C NMR (75 MHz, $CDCl_3$); *2-(4-Benzylpiperazin-1-yl)ethyl (Z)-N-phenylmorpholine-4-carbimidothioate* **3j**

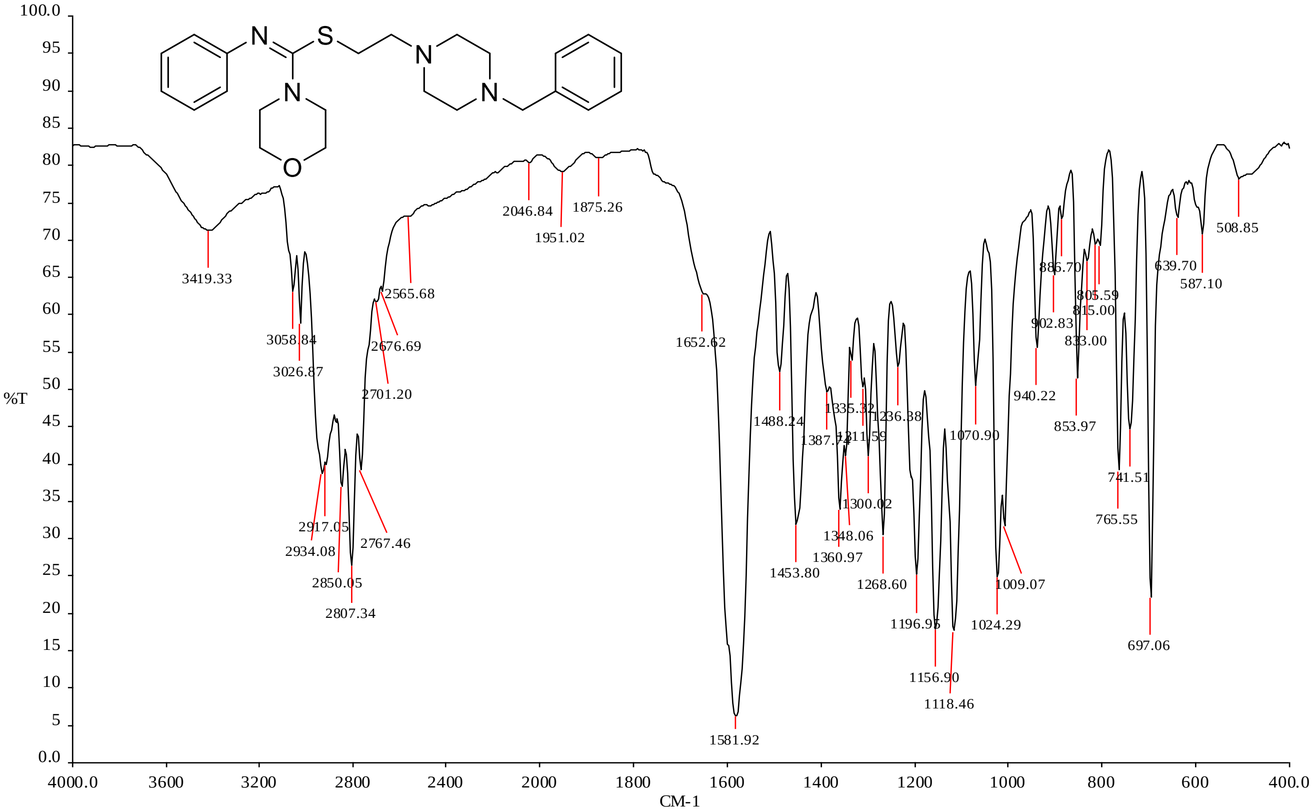

Mr. Moradi **Sample: D 11**

Acquired : 3 Jan 2007 7:20 using AcqMethod f1.M
Instrument : MSD
Sample Name: D11
Misc Info :
Vial Number: 1

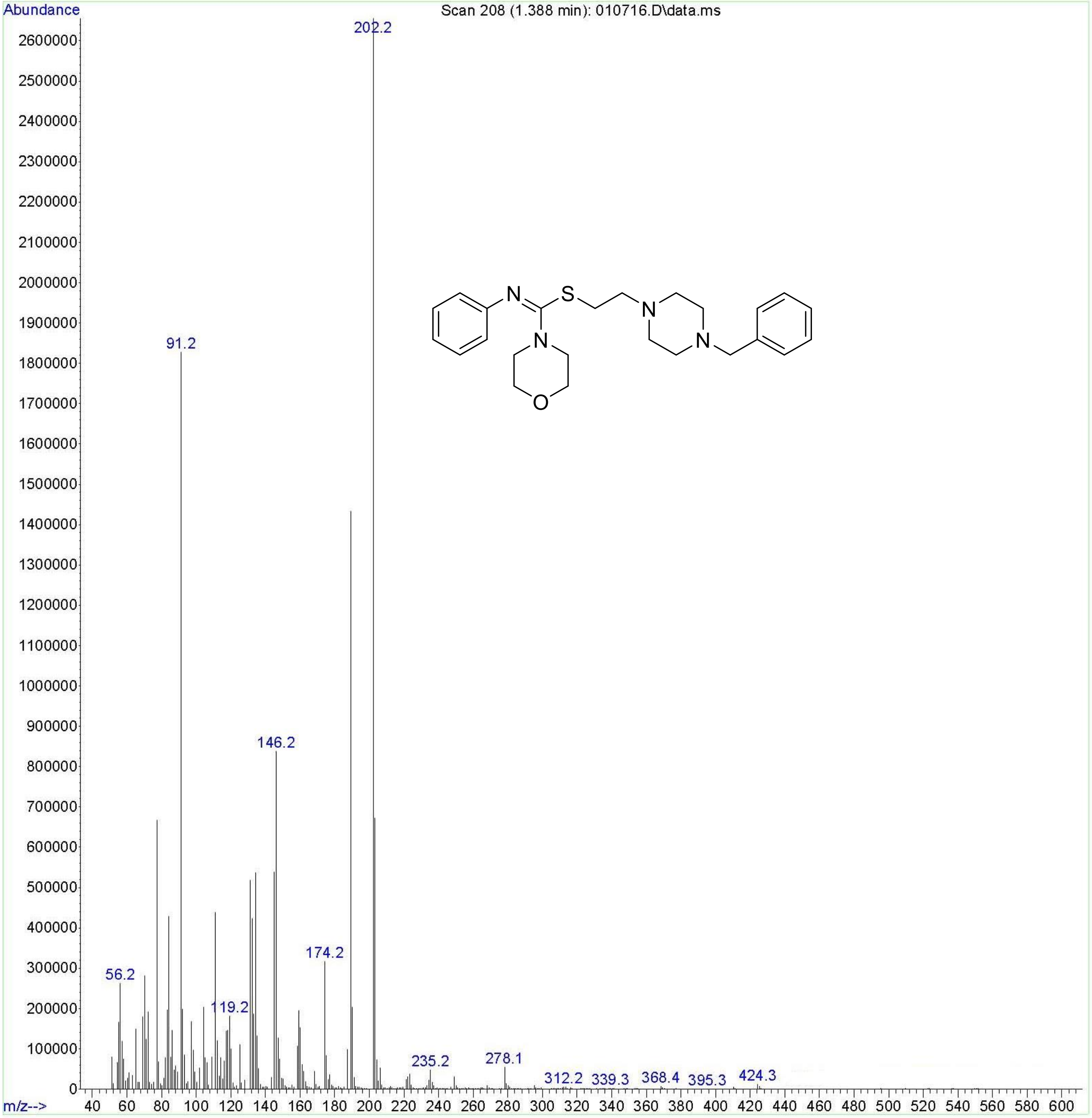

Supplement: RA-013-D3RA06678A-s001 [file RA-013-D3RA06678A-s001.pdf]
